# Perivascular cell‐derived extracellular vesicles stimulate colorectal cancer revascularization after withdrawal of antiangiogenic drugs

**DOI:** 10.1002/jev2.12096

**Published:** 2021-05-21

**Authors:** Maohua Huang, Minfeng Chen, Ming Qi, Geni Ye, Jinghua Pan, Changzheng Shi, Yunlong Yang, Luyu Zhao, Xukai Mo, Yiran Zhang, Yong Li, Jincheng Zhong, Weijin Lu, Xiaobo Li, Jiayan Zhang, Jinrong Lin, Liangping Luo, Tongzheng Liu, Patrick Ming‐Kuen Tang, An Hong, Yihai Cao, Wencai Ye, Dongmei Zhang

**Affiliations:** ^1^ College of Pharmacy Jinan University Guangzhou China; ^2^ Department of General Surgery the First Affiliated Hospital of Jinan University Guangzhou China; ^3^ Guangzhou Key Laboratory of Molecular and Functional Imaging for Clinical Translation the First Affiliated Hospital of Jinan University Guangzhou China; ^4^ Department of Cellular and Genetic Medicine School of Basic Medical Sciences Fudan University Shanghai China; ^5^ Department of Obstetrics the First Affiliated Hospital of Jinan University Guangzhou China; ^6^ Department of Anatomical and Cellular Pathology Prince of Wales Hospital The Chinese University of Hong Kong Sha Tin Hong Kong; ^7^ Department of Cell Biology Jinan University Guangzhou China; ^8^ Department of Microbiology Tumor and Cell Biology Karolinska Institute Stockholm Sweden

**Keywords:** antiangiogenic therapy, endothelial progenitor cell, extracellular vesicle, perivascular cell, tyrosine kinase inhibitor

## Abstract

Antiangiogenic tyrosine kinase inhibitors (AA‐TKIs) have become a promising therapeutic strategy for colorectal cancer (CRC). In clinical practice, a significant proportion of cancer patients temporarily discontinue AA‐TKI treatment due to recurrent toxicities, economic burden or acquired resistance. However, AA‐TKI therapy withdrawal‐induced tumour revascularization frequently occurs, hampering the clinical application of AA‐TKIs. Here, this study demonstrates that tumour perivascular cells mediate tumour revascularization after withdrawal of AA‐TKI therapy. Pharmacological inhibition and genetic ablation of perivascular cells largely attenuate the rebound effect of CRC vascularization in the AA‐TKI cessation experimental settings. Mechanistically, tumour perivascular cell‐derived extracellular vehicles (TPC‐EVs) contain Gas6 that instigates the recruitment of endothelial progenitor cells (EPCs) for tumour revascularization via activating the Axl pathway. Gas6 silence and an Axl inhibitor markedly inhibit tumour revascularization by impairing EPC recruitment. Consequently, combination therapy of regorafenib with the Axl inhibitor improves overall survival in mice metastatic CRC model by inhibiting tumour growth. Together, these data shed new mechanistic insights into perivascular cells in off‐AA‐TKI‐induced tumour revascularization and indicate that blocking the Axl signalling may provide an attractive anticancer approach for sustaining long‐lasting angiostatic effects to improve the therapeutic outcomes of antiangiogenic drugs in CRC.

## 1 INTRODUCTION

Antiangiogenic therapy is widely used for treating various tumours, including colorectal cancer (CRC) (Tampellini et al., 2016). Antiangiogenic tyrosine kinase inhibitors (AA‐TKIs), such as regorafenib and axitinib, inhibit tumour angiogenesis by suppressing the vascular endothelial growth factor (VEGF)/VEGF receptor (VEGFR) signalling pathway (Grothey et al., 2013; Sharma et al., 2010). These drugs improve overall survival (OS) and disease‐free survival (DFS) in patients with metastatic CRC undergoing monotherapy and combination therapy regimens (Grothey et al., 2013; Sharma et al., 2010; Tampellini et al., 2016). Despite their initial responses, most patients frequently showed rapid revascularization, tumour regrowth, and metastasis after cessation of AA‐TKI therapy, which is one of the key obstacles that limit its clinical application (Ebos & Kerbel, 2011; Ebos et al., 2009; Haemmerle et al., 2016; Sounni et al., 2014). AA‐TKI treatment induces extensive endothelial cell loss in tumour vessels, leading to the formation of empty sleeves of the basement membrane (BM) with persistent perivascular cell coverage (Mancuso et al., 2006; Runge et al., 2014). The perivascular cell‐BM supportive structure serves as a scaffold for rapid revascularization after the withdrawal of antiangiogenic therapy (Abdullah & Perez‐Soler, 2012; Mancuso et al., 2006). However, the contribution of tumour perivascular cells to drug cessation‐induced tumour revascularization remains uncharted.

Perivascular cells that embedded into the BM of blood vessels play a critical role in vascular formation and stabilization by interacting with endothelial cells (Armulik et al., 2005). Perivascular cells induce VEGFA production to activate survival signalling in endothelial cells through an autocrine mechanism, which protects tumour vessels from antiangiogenic drugs (Franco et al., 2011). In addition, perivascular cells participate in BM formation and promote angiogenesis through the production of HSP47 (Hosono et al., 2017). The recruitment of endothelial progenitor cells (EPCs) into tumour tissues may involve in tumour vasculogenesis or the repair of injured tumour vessels (Shaked, 2006; Taylor et al., 2012). Cancer cells, together with existing endothelial cells, secrete various chemokines and cytokines to recruit EPCs to tumours (De La Puente et al., 2013; De Palma et al., 2017). However, the role of perivascular cells in recruiting EPCs into tumours is unclear.

Extracellular vehicles (EVs), including microvesicles and exosomes, are extracellular membrane vesicles with diameters of variable sizes and secreted by most cells. EVs contain various non‐coding RNAs and proteins, which serve as important signalling entities in mediating cellular communications between diverse cell types (Mashouri et al., 2019; Shao et al., 2018). Cancer cell‐ and stromal cell‐derived EVs have been confirmed to participate in the regulation of tumour growth, metastasis, and angiogenesis (Kalluri, 2016; Ludwig & Whiteside, 2018; Mashouri et al., 2019). Antiangiogenic therapy induces the release of VEGF‐enriched EVs from endothelial cells to promote tumour vasculogenesis (Zeng et al., 2019). In addition, perivascular cells are capable of secreting EVs that can regulate angiogenesis and vascular functions. For example, the cerebral perivascular cell‐derived EVs improve microcirculation after spinal cord injury in mice (Yuan et al., 2019). Circular RNA‐cPWWP2A is upregulated in perivascular cell‐derived EVs in response to diabetes‐related stress, which alleviates diabetes‐induced retinal vascular dysfunction (Liu et al., 2019). However, the contents and functions of tumour perivascular cell‐derived EVs (TPC‐EVs) are unknown.

The aim of this study is to investigate the mechanisms by which tumour perivascular cells confer CRC revascularization after withdrawal of regorafenib or axitinib treatment. Herein, we employ human CRC xenograft models and mouse CRC allograft models that recapitulate initial clinical response and subsequent withdrawal of AA‐TKI therapy to induce a rebound phenomenon of tumour vasculature. Our results indicate that TPC‐EVs recruit EPCs into tumours and contribute to CRC revascularization through the EV‐Gas6/Axl pathway.

## 2 MATERIALS AND METHODS

### 2.1 Ethics statement

The clinical specimens used in this study were approved by the Review Board of The First Affiliated Hospital of Jinan University and written informed consent was received from participants prior to their inclusion in the study. All animal studies were conducted with the approval of the Laboratory Animal Ethics Committee of Jinan University (approve number: 2018107‐02) and adhered to the NIH Guide for the Care and Use of Laboratory Animals.

### 2.2 Isolation and culture of tumour perivascular cells

Tumour perivascular cells (TPC) were isolated from human colorectal cancer tissues that were collected after surgical excision at the First Affiliated Hospital of Jinan University (Supplementary Table 1). The tissues were washed with pre‐chilled Hank's balanced salt solution (HBSS, Gibco) to remove surface blood, adipose tissues, and tissue fragments. Tumour vessels were separated and minced in pre‐chilled HBSS before endothelial cells were removed under a dissecting microscope. The rinsed vessel pieces were digested with tumour dissociation kit (Miltenyi Biotec) and then performed with gentleMACS Dissociators (Miltenyi Biotec) according to the manufacturer's instructions. The dissociated cells were washed with phosphate buffer saline (PBS, HyClone) twice, followed by culture in Pericyte Medium (PM, ScienCell) containing 2% fetal bovine serum (FBS, ExCell Bio), 1% Pericyte Growth Supplement (PGS), and 1% penicillin‐streptomycin. The cells were maintained in the atmosphere at 37℃ with 5% CO_2_. Adherent cells were collected and identified with transmission electron microscope (TEM) analysis, immunofluorescence assay, and flow cytometry (FCM) analysis.

### 2.3 Isolation and culture of EPCs

Preparation of EPCs was performed as previously described with some modifications (Maeng et al., 2009). Briefly, mononuclear cells were isolated from human umbilical cord blood samples by Histopaque‐1077 and 1199 (Sigma) density gradient centrifugation for 30 min at 700 g, followed by two washes in PBS. Approximately 1 × 10^6^ mononuclear cells were seeded in fibronectin‐coated 6‐well plates and maintained in the culture system containing EBM‐2 Basal Medium (Lonza), EGM‐2 SingleQuots Supplements (Lonza) and 1% penicillin‐streptomycin (Gibco). The supplements consisted of FBS, hydrocortisone, ascorbic acid, GA‐1000, human fibroblast growth factor‐B, VEGF, insulin‐like growth factor, epidermal growth factor, and heparin. Following culture for 3 days, non‐adherent cells were removed. Fresh medium was applied and adherent cells were further cultured for 4 days. Phenotypic analysis of the cells was performed at day 0, 4, 7, 14 and 28. EPC identification and purity (over 95%) were determined by Dil‐labeled acetyl low‐density lipoprotein (Dil‐ac‐LDL, Molecular Probes) uptake capacity, double staining with Dil‐ac‐LDL and FITC‐labelled *Ulex europaeus* lectin I (FITC‐UEA‐1, Vector Laboratories), immunofluorescence staining with CD34, CD133 (Abcam), CD31 (R&D system), and VE‐cadherin (Cell Signaling Technology), and flow cytometry analysis of CXCR4, CD31, CD309 (VEGFR2), and CD45 (BioLegend).

### 2.4 Cells and cell culture

Human umbilical vein endothelial cells (HUVECs) were obtained from ScienCell and cultured in Endothelial Cell Medium (ECM, ScienCell) containing 5% FBS, 1% Endothelial Cell Growth Supplement (ECGS), and 1% penicillin‐ streptomycin. TPCs, EPCs, and HUVECs were used at passage 2 to 6. Human colorectal cancer HT‐29 and HCT116 cells (ATCC), and mouse colorectal cancer Colon26 cells (JCRB Cell Bank) were cultured in DMEM (Invitrogen) supplemented with 10% FBS and 1% penicillin‐streptomycin (HyClone). All cells were cultured at 37°C in a humidified atmosphere containing 5% CO_2_. All cell lines were negative for mycoplasma tested using the Mycoplasma Detection Set (M&C Gene Technology). For EV treatment experiments, FBS was replaced with exosome‐depleted FBS (edFBS, Cat. EXO‐FBS‐50A‐1, System Biosciences).

### 2.5 Separation, characterization and quantification of TPC‐EVs

The separation, characterization, and quantification of TPC‐EVs were performed in compliance with the Minimal information for studies of extracellular vesicles 2018 (MISEV2018) guidelines proposed by the International Society for Extracellular Vesicles (ISEV) (Théry et al., 2018). The characterization and quantification of TPC‐EVs were determined by TEM analysis, Western blotting analysis, and nanoparticle tracking analysis (NTA).

For the separation of TPC‐EVs, 1 × 10^6^ TPCs (passage 4 to 5) were seeded in 150 cm^2^ cell culture flasks and grown to an approximate 75% confluency. The culture medium was removed and replaced by serum‐free PM containing 1% PGS and 1% penicillin‐streptomycin. After a 48‐h culture, the medium (approximately 15 ml/per flask) was collected and centrifuged at 2, 000 × g at 4℃ for 10 min to remove dead cells. The supernatants were harvested and further centrifuged for 10, 000 × g at 4℃ for 30 min to remove cellular debris, followed by sterile filtration with 0.22 μm filters (Millipore). Next, the TPC‐EVs were separated using ExoQuick‐TC Exosome Precipitation Solution (EXOTC50A‐1, System Biosciences). The solution was added to the filtered supernatants at a ratio of 1:5 and the mixture was refrigerated at 4℃ for over 36 h. Subsequently, the samples were first centrifuged at 1, 500 × g at room temperature for 30 min and then the supernatants were removed, followed by another centrifugation at 1, 500 × g at room temperature for 5 min. The TPC‐EV pellets were collected and resuspended in 100 μl PBS and stored at ‐80℃ for further experiments.

The morphology of TPC‐EVs was assessed by TEM analysis as previously described (Gong et al., 2020). For quantification of TPC‐EVs, the concentration of TPC‐EVs was measured in terms of their protein content, which was determined by a BCA Protein Assay Kit (Thermo Fisher Scientific). Briefly, 10 μl of TPC‐EV samples without detergent treatment were loaded into a 96‐well plate and 100 μl of the BCA solution was added. Then, the plate was incubated at 65℃ for 15 min in the dark and the absorbances were detected by a microplate reader at a wavelength of 595 nm. A standard curve was used to determine the protein concentration of TPC‐EVs. The particle size distribution and particle concentration (particles/ml) of TPC‐EVs were performed with NTA using NanoSight LM10 equipped with a 405‐nm laser (Malvern Instruments) and the data were analyzed with the NTA software (NTA 3.2 Dev Build 3.2.16. Malvern Instruments). The purity of EVs was determined by the ratios of proteins: particles as previously reported (Webber & Clayton, 2013). The expression of CD63, HSPA8/HSC70, Syntenin‐1, and GM310 in TPC‐EVs were assessed by Western blotting analysis.

### 2.6 Western blotting analysis

Briefly, cells were harvested and lysed with RIPA buffer (Thermo Scientific) containing protease and phosphatase inhibitors (Roche). Then, protein levels were detected by Western blotting analysis as previously described (Lei et al., 2018). The following antibodies were used: CD63 (ab134045, Abcam), HSPA8/HSC70 (8444, Cell Singling Technology), Syntenin‐1 (ab133267, Abcam), GM130 (12480, Cell Singling Technology), p‐Axl (Y779) (AF2228, R&D system), Axl (8661, Cell Singling Technology), p‐Akt (Ser473) (4060, Cell Singling Technology), Akt (4691, Cell Singling Technology), p‐Erk1/2 (Thr202/Tyr204) (4270, Cell Singling Technology), Erk1/2 (4695, Cell Singling Technology), β‐actin (4970, Cell Singling Technology), and Gas6 (67202, Cell Singling Technology). Anti‐rabbit IgG, HRP‐linked antibody (7074, Cell Signaling Technology) was used as secondary antibody.

### 2.7 Proteomic analysis of TPC‐EVs

Proteome profiling of TPC‐EVs was performed by Shanghai Applied Protein Technology Co. Ltd. Mass spectrometry data were obtained using a LC‐MS/MS (nanoLC‐QE) system equipped with Q Exactive (Thermo Fisher Scientific) coupled to an Easy‐nLC 1000 (Thermo Fisher Scientific). The protein isolation and identification, filter‐aided sample preparation (FASP), MS data acquisition, and data processing of perivascular cell‐derived extracellular vesicles were performed according to a prior study with some modifications (Gong et al., 2020).

In brief, TPC‐EV samples (50 μl, obtained from 100 ml TPC culture medium) were quantified and sample with 20 μg proteins were performed with SDS‐PAGE separation, followed by filter‐aided sample preparation (FASP digestion).

Next, the tryptic fragments were loaded onto a reversed phase Trap column (Thermo Fisher Scientific, Acclaim PepMap100, C18, 5 μm, 100 μm × 2 cm) with nanoViper fittings, and separated by a reversed phase analytical column (Thermo Fisher Scientific Easy Column, C18, 3 μm, 100 mm × 75 μm). The mobile phases were buffer A (0.1% FA) and B (0.1% FA in 84% ACN), and the peptides were eluted with a linear gradient for buffer B (0‐35% for 50 min, 35–100% for 5 min, and maintained at 100% for 5 min) at a flow rate of 300 nl/min controlled by IntelliFlow technology.

For LC‐MS/MS analysis, it was performed on a Q Exactive, which was run with peptide recognition mode enabled. The overall running time of analysis was 120 min. The mass spectrometer was operated in positive ion mode. After MS2 scan, the top 20 abundant precursor ions from the survey scans (mass‐to‐charge, m/z = 300 ‐ 1,800) were chosen for higher‐energy collisional dissociation (HCD) fragmentation. The automatic gain control (AGC) target was set to 3e6, maximum inject time was 10 ms, and dynamic exclusion duration was 40 s. The resolution of survey scan was set to 70, 000 at 200 m/z, while the resolution for HCD spectra was adjusted to 17, 500 at 200 m/z and isolation width was 2 m/z. The normalized collision energy was 30 eV and the underfill ration that specified the minimum percentage of the target value likely to be reached at maximum fill time, was defined as 0.1%.

Raw data files were then converted to MGF format by Proteome Discoverer software (version 1.4, Thermo Fisher Scientific). Then proteins were identified with Mascot algorithm (version 2.2, Matrix Science) by match peptides with data in the UniProt database (173343 entries, downloaded on 14 October, 2019). The following options were used for protein identification: Enzyme = trypsin, Fixed modification = Carbamidomethyl (C), Dynamical modifications = Oxidation (M), Max Missed Cleavages = 2.

### 2.8 Establishment of human colorectal cancer xenografts and treatments

HT‐29 or HCT116 cells (2 × 10^6^ cells/ml) suspended in diluted Matrigel (diluted with PBS at a ratio of 1:3) were inoculated subcutaneously in the flank of 6‐8‐week‐old female BALB/c nude mice (Vital River Laboratory Animal Technology Co., Beijing, China). Antiangiogenic TKIs regorafenib and axitinib (TargetMol) suspended in 0.5% CMC‐Na solution (Selleck Chemicals) were used for *in vivo* studies. When tumours grew to about 300 mm^3^, mice were grouped and treated with anti‐angiogenic TKIs. (1) For the tumour revascularization study, tumour‐bearing mice were treated with: (a) vehicle [0.5% sodium carboxymethylcellulose (CMC‐Na) in water, oral gavage, qd)]; (b) regorafenib (30 mg/kg, oral, qd), axitinib (25 mg/kg, oral, qd) for 7 days; or (c) regorafenib or axitinib for 7 days followed by a period without treatment lasting 7 days. (2) For the role of TPC‐EVs in tumour revascularization, tumour‐bearing mice received the treatment of regorafenib or axitinib for 7 days were then treated with: (a) tail vein injection of vehicle for 4 days; (b) tail vein injection of TPC‐EVs or TPC‐EV‐(siNC) (100 μg) for 4 days; or (c) tail vein injection of TPC‐EV‐(siGas6) (100 μg) for 4 days. (3) For the role of the Gas6/Axl pathway in tumour revascularization, tumour‐bearing mice treated with regorafenib or axitinib for 7 days then received: (a) vehicle, oral gavage of 0.5% CMC‐Na/0.1 % Tween‐80 in water for 7 days; or (b) oral gavage of R428 (50 mg/kg, bid) for 7 days. For the role of tumour perivascular cells in tumour revascularization, tumour‐bearing mice were treated with regorafenib or axitinib in combination with imatinib (45 mg/kg, intraperitoneal injection, bid, TargetMol^®^) for 7 days or anti‐mouse PDGFRβ/CD140b neutralizing antibody (75 μg/mouse, intraperitoneal injection, Biolegend) every other day for 5 times, followed by a period without treatment lasting 7 days. Tumour tissues were collected for immunofluorescence assay and FCM analysis.

### 2.9 Establishment of mice colorectal cancer allografts and treatments

Breeding pair of NG2‐thymidine kinase (tk) mice was a gift from Dr. Raghu Kalluri (University of Texas MD Anderson Cancer Center) (Cooke et al., 2012). The stain was maintained by breeding hemizygous males with FVB wild‐type (WT) females. Mice were backcrossed to BALB/c background for more than 10 generations. Mouse colorectal cancer Colon26 cells (5 × 10^5^ cells) were resuspended in serum‐free medium and subcutaneously implanted into the flank of 6‐to‐12‐week‐old female NG2‐tk mice and WT mice. When average tumour size reached approximately 200 mm^3^ (about 12 days post tumour cell inoculation), NG2‐tk mice and WT mice received daily intraperitoneal injections with 50 mg/kg ganciclovir (GCV, diluted in 0.9% sodium chloride injection). Regorafenib (20 mg/kg) was given daily for 7 days by intragastric administration, followed by a period without treatment lasting 5 days. Then, tumour tissues were collected for immunofluorescence assay and flow cytometry analysis.

### 2.10 Preclinical model of colorectal cancer liver metastases

The establishment of colorectal cancer liver metastasis models was performed as previously described (Frentzas et al., 2016). HTC116 cells were resuspended in diluted Matrigel at a density of 1 × 10^7^ cells/ml and implanted into the liver of 6–8 weeks old female BALB/c nude mice by laparotomy under general anaesthesia with inhaled isoflurane. The left lobe of the liver was injected with 3 × 10^5^ cells in a volume of about 30 μl by a 29‐gauge needle. Then, the liver was returned to the peritoneal cavity and the wound was sutured. The tumour was established for over 15 days. For overall survival experiments, mice were treated with vehicle (0.5% CMC‐Na in water, oral gavage, qd), regorafenib (30 mg/kg, oral gavage, qd), R428 (50 mg/kg, oral gavage, qd), or regorafenib plus R428 every day for 14 days, followed by a period without treatment until necropsy. For tumour vascular analysis, mice received the indicated treatments for 14 days, and then tumour tissues were collected for immunofluorescence assay.

### 2.11 Immunofluorescence assay

To analyze the surface maker of EPCs and TPCs, cells were seeded in glass‐bottomed dishes and cultured overnight. Then, the cells were fixed with 4% PFA, blocked with 5% BSA and incubated with primary antibodies overnight at 4℃. Then, the cells were incubated with secondary antibodies at room temperature in the dark for 1 h followed by DAPI (Sigma) staining. For tissue immunofluorescence staining, tumour tissues were removed, fixed with 4% PFA overnight, dehydrated in 30% sucrose/PBS solution and then embedded in OCT (Sakura). The 8‐μm‐thick cryosections were blocked, permeabilized, and then incubated with anti‐CD31 (AF3628, R&D Systems), anti‐PDGFRβ (CD140b) (14‐1402‐82, eBioscience), anti‐NG2 (AB5320, Chemicon), anti‐phospho‐Histone H3 (Ser10) (3458, Cell Signaling Technology), or anti‐α‐SMA‐Alexa Fluor 488 (34105, Cell Signaling Technology) antibodies overnight at 4℃, followed by incubation with corresponding secondary antibodies (Invitrogen) at room temperature in the dark for 1 h. DAPI was used for nuclear staining. The images were photographed under a Zeiss LSM 800 confocal microscope (Zeiss).

### 2.12 In vivo magnetic resonance imaging (MRI) analysis

In vivo MRI analysis was performed as previously described with some modifications (Busato et al., 2016; Chen et al., 2017). MRI images were acquired using a 9.4 T animal MRI (BioSpec 94/30 USR, Bruker, Ettlingen, Germany). The BALB/c nude mice were anesthetized with 2%‐4% Isoflurane and 96%‐98% oxygen at a flow rate of 1 ‐ 3 L/min. They were placed in a prone position on a heated bed, and then inserted into a 3.5‐cm internal diameter birdcage radiofrequency coil. To perform in vivo MRI analysis, high‐resolution coronal T2‐weighted (T2W) fast spin‐echo images were acquired using the rapid acquisition with refocused echoes (RARE) sequence (TR = 2500 ms, TE_eff_ = 22 ms, FOV = 30.0 × 30.0 mm^2^, Image Size = 256 × 256, Slice Thickness = 0.7 mm, Slice Gap = 0 mm, Rare Factor = 4, NEX = 4). Then, USPIO‐loaded TPC‐EVs were intravenously injected into mice, and USPIO‐loaded TPC‐EV incorporation into the bone marrow was determined before and at 24 h after TPC‐EV injection.

### 2.13 Flow cytometry analysis

For the identification of EPCs and TPCs, adherent cells were dissociated with Cellstripper buffer (Corning) and washed twice with Flow Cytometry Staining Buffer (Invitrogen). Then EPCs were incubated with phycoerythrin (PE)‐conjugated anti‐CXCR4 (clone 12G5, BioLegend), anti‐CD309/VEGFR2 (clone 7D4‐6, BioLegend), anti‐CD31 (clone WM59, BioLegend), anti‐CD45 (clone 2D1, BioLegend), and anti‐Axl (clone DS7HAXL, Invitrogen) antibodies. TPCs were incubated with PE‐anti‐α‐SMA (clone 1A4, R&D Systems), anti‐FAPα (clone 427819, R&D Systems), anti‐PDGFRβ/CD140b (clone APB5, BioLegend), anti‐CD31, anti‐AN2/NG2 (clone 1E6.4, Miltenyi Biotec), and anti‐CD324/E‐cadherin (clone 67A4, Miltenyi Biotec) antibodies. PE‐conjugated mouse IgG isotype control was used as a negative control. For the analysis of EPCs recruited into tumours, tumour tissues were minced, digested with 1 mg/ml collagenase type I (Worthington) and 50 U/ml hyaluronidase (Worthington) in a 37℃ incubator for 1 h. The digested tissues were filtered with a 30‐μm cell strainer and single cells were washed with PBS twice. The single cells were blocked by incubation with an anti‐mouse CD16/CD32 antibody (clone 93, BioLegend), followed by staining with rat anti‐mouse CD309/VEGFR2‐PE (clone Avas12a1, Invitrogen), anti‐CD45‐PerCP (clone 30‐F11, BioLegend), and anti‐CD117/c‐kit‐APC (clone 2B8, BioLegend) antibodies on ice in the dark for 1 h. DAPI was added to the stained tubes to exclude dead cells before flow cytometry analysis. Flow cytometry analysis was performed using a FACS Canto system (BD Biosciences), and the data were analyzed by FlowJo software (Tree Star).

### 2.14 Transwell migration assay

Cell migration assay was performed with the Transwell system (Corning Costar) containing polycarbonate filters (diameter; 6.5‐mm) with a pore size of 8 μm. EPCs (4 × 10^4^) were resuspended in 100 μl of EBM‐2 basal medium and seeded into the upper filters, and then, 500 μl of EBM‐2/5% edFBS medium containing TPC‐EVs were added to the lower chamber. After a 24‐h incubation, the non‐migrating cells in the top of the upper filters were removed, and the migrated cells were fixed with 4% paraformaldehyde (PFA, Sigma) for 15 min. After staining with 0.1% crystal violet solution (Sigma) for 30 min, cells were photographed and the number of migrated cells was quantified with Image‐Pro Plus 6.0 software.

### 2.15 Cell adhesion assay

The cell adhesion assay was performed as previously described (Kim et al., 2015; Maeng et al., 2009). The 96‐well plates were pre‐coated with 50 μg/ml human fibronectin (Corning) overnight at 4℃ or with confluent HUVECs overnight at 37℃. EPCs (2 × 10^4^) were resuspended in 100 μl of EBM‐2 basal medium and then seeded into pre‐coated 96‐well plates with or without TPC‐EVs, and incubated for 30 min at 37℃. The non‐adherent cells were removed, and the adherent cells were washed twice with PBS. For the fibronectin adhesion assay, adherent cells were fixed with 4% PFA, stained with 0.1% crystal violet solution and photographed with an inverted microscope. For the HUVEC adhesion assay, EPCs were pre‐stained with PKH26 and the images of adherent cells were captured with an inverted fluorescence microscope. Adhesion was quantified in triplicates by counting adherent cells in five randomly selected fields per well.

### 2.16 Cell proliferation and viability assay

Cell proliferation was detected by an EdU cell proliferation assay (GeneCopoeia) and cell viability was measured by an MTT assay (Sigma). For the proliferation assay, 2.5 × 10^3^ EPCs were seeded in 96‐well plates and cultured overnight, and then the medium was replaced and incubated with or without TPC‐EVs for 48 h. The cells were analyzed according to the protocol of the EdU cell proliferation kit. The number of EdU‐positive cells was calculated using Image‐Pro Plus 6.0 software (Media Cybernetics). For the viability assay, 5 × 10^3^ EPCs were seeded in 96‐well plates and cultured overnight, and then the medium was replaced with serum‐free EBM‐2 and incubated with or without TPC‐EVs for 24 h. The medium was discarded and the cells were incubated with 5 mg/ml MTT for 2 h at 37℃. Then, formazan was dissolved with 100 μl of DMSO (Sigma) and the absorbance was measured with a microplate reader at a test wavelength of 595 nm.

### 2.17 In vitro neovessel incorporation assay

HUVECs were used for tube formation assays as previously described (Kim et al., 2015). EPCs were stained with PKH26 (Sigma) and washed with PBS. The 96‐well plates were pre‐coated with 40 μl growth factor‐reduced Matrigel (Corning) for 1 h at 37℃, and then 0.5 × 10^4^ EPCs mixed with 2.5 × 10^4^ HUVECs in EBM‐2/5% edFBS were seeded on pre‐coated plates in the presence or absence of TPC‐EVs for 4 h. The images of EPCs incorporated into new‐formed tubes were photographed by fluorescence microscopy. The number of incorporated EPCs, tube number, and node number of the neovessel network were quantified using Image‐Pro Plus 6.0 software.

### 2.18 Real‐time polymerase chain reaction (RT‐PCR) assay

EPCs with 60% confluence were treated with or without TPC‐EVs for 24 h, and then total RNA was extracted by the Total RNA Kit I (Omega Bio‐Tek) according to the manufacturer's protocol. Reverse transcription of total RNA and RT‐PCR were performed according to the standard operating procedures. The relative mRNA expression levels were normalized to that of the housekeeping gene *ACTB*, and the results are presented as the fold change compared to the vehicle group. Briefly, the 2^–ΔΔCT^ method was used to calculate the relative expression of each mRNA. The primers sequences used are listed in Supplementary Table 2.

### 2.19 Transfection of small interfering RNA

For gene knockdown experiments, cells were transfected with the indicated small interfering RNA (siRNA) duplexes or a negative control siRNA using Lipofectamine 3000 (Invitrogen). The siRNA sequences are shown in Supplementary Table 3. After a 6‐h transfection, the medium was replenished and the cells were cultured in fresh medium for 24 h, followed by the indicated treatments.

### 2.20 Ultrasound imaging analysis

The tumour burden was monitored by a high‐frequency ultrasound imaging system (Toshiba, Aplio 500) according to a prior report (Jung et al., 2017). Ultrasound imaging analysis was performed in tumour‐bearing mice under general anaesthesia with inhaled isoflurane. Liver tumours were identified as a low echoic mass and images were acquired with the Toshiba PLT‐604AT Ultrasound Probe. Long diameter (LD) and shorted diameter (SD) were obtained from the images at days 15 and 28 after tumour implantation.

### 2.21 Statistical analysis

All in vitro experiments were performed at least three independent times. Statistical analyses were performed with GraphPad Prism 8.0 software (GraphPad Software). Data are presented as mean ± standard errors of the mean (SEM). Significant differences between two groups were evaluated using unpaired two‐tailed *t*‐test and significant differences between more than two groups were evaluated using one‐way ANOVA and Tukey's multiple comparison test. *P* values* <* 0.05 were considered statistically significant.

## 3 RESULTS

### 3.1 Pharmacological inhibition of perivascular cells prevents off‐AA‐TKI‐triggered revascularization

**FIGURE 1:**
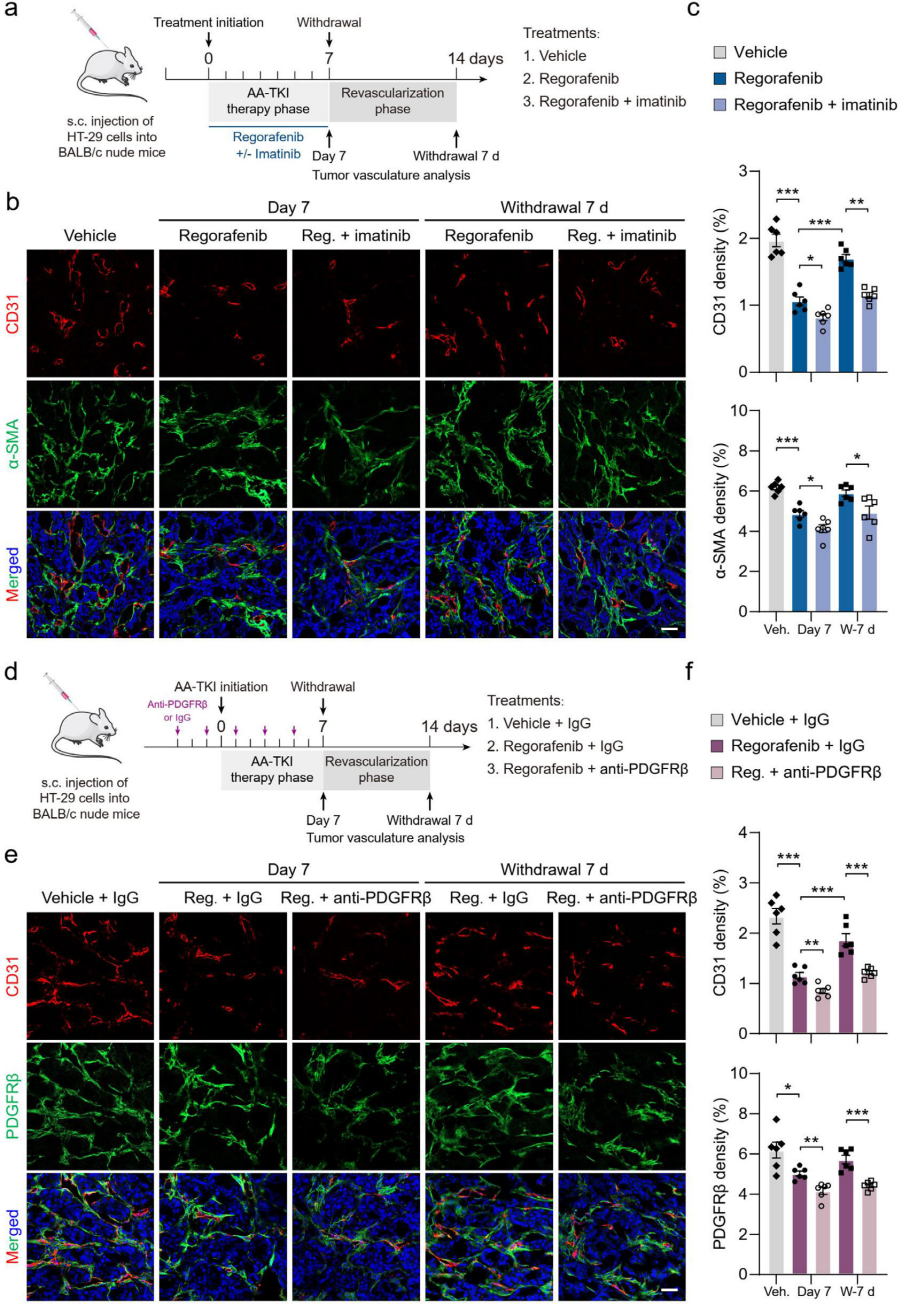
AA‐TKI withdrawal‐induced tumor revascularization is reduced after depletion of α‐SMA^+^ or PDGFRβ^+^ perivascular cells. (a) Therapeutic schedule for treatment with vehicle, regorafenib, and regorafenib + imatinib. Tumor vasculatures were analyzed at days 7 (Day 7) and 14 (withdrawal 7 d) after the indicated treatments. Withdrawal 7 d, 7 days after AA‐TKI withdrawal. (b) Representative images of CD31^+^ (red) endothelial cell, α‐SMA^+^ (green) perivascular cell immunostaining, and DAPI (blue) for nuclear staining. Reg. regorafenib. (c) Quantification of microvessel density (CD31 density) and perivascular cell number (α‐SMA density) in HT‐29 tumors (n = 6). Veh., vehicle. W‐7 d, 7 days after AA‐TKI withdrawal. (d) Therapeutic schedule for treatment with vehicle + IgG, regorafenib + IgG, and regorafenib + anti‐PDGFRβ. Tumor vasculatures were analyzed at days 7 (Day 7) and 14 (withdrawal 7 d) after the indicated treatments. (e) Representative images of CD31^+^ (red) endothelial cell, PDGFRβ^+^ (green) perivascular cell immunostaining, and DAPI (blue) for nuclear staining. (f) Quantification of microvessel density (CD31 density) and perivascular cell number (PDGFRβ density) in HT‐29 tumors (n = 6). Data are present as mean ± SEM. Scale bars, 50 μm. ^*^ *P* < 0.05, ^**^ *P* < 0.01, and ^***^ *P* < 0.001

Drug withdrawal‐triggered revascularization critically curtails the clinical applicability of antiangiogenic therapy. However, the mechanisms of revascularization remain largely unknown. In this study, we aim to tackle this critical issue using clinically relevant animal tumour models. CRC (HT‐29)‐bearing BALB/c nude mice tumours were treated with AA‐TKIs (regorafenib or axitinib) for 7 days (AA‐TKI therapy phase), followed by a 7‐day off‐drug phase to allow tumour revascularization (revascularization phase). Tumour microvasculature was analyzed by immunohistochemistry at day 7 (7 days post‐therapy initiation, Day 7) and day 14 (AA‐TKI therapy withdrawal for 7 days, withdrawal 7 d) (Figure 1a and Figure S1a). CD31^+^ vessels of HT‐29 xenograft tumours in the vehicle groups were abundant, tortuous, and variable in diameter and attached with α‐SMA^+^ perivascular cells (Mancuso et al., 2006; Ruan et al., 2013) (Figure 1b and c). Similar to the findings in a previous report (Mancuso et al., 2006), 7 days of regorafenib or axitinib therapy induced a marked decrease of CD31 density, but showed a marginal effect on α‐SMA^+^ perivascular cells (Figure 1b and c, and Figure S1b and c), which indicated that AA‐TKI therapies often led to residual perivascular cells in tumour tissues. After treatment withdrawal for 7 days (withdrawal 7 d), tumours became rapidly revascularized, as indicated by a more than 1.5‐fold increase in CD31 microvessel density compared with tumours formed following AA‐TKI treatment for 7 days (Figure 1b and c, and Figure S1b and c).

To assess the effect of perivascular cells on drug withdrawal‐related tumour revascularization, imatinib was used to deplete tumour perivascular cells (Ruan et al., 2013). As expected, compared to the tumours observed after 7 days of regorafenib or axitinib monotherapy, the combination of regorafenib or axitinib with imatinib induced a sharp reduction in the number of α‐SMA^+^ tumour perivascular cells. In the revascularization phase, the combination treatment with imatinib significantly attenuated tumour revascularization and tumours became less revascularized, relative to tumours obtained following treatment with regorafenib or axitinib alone (Figure 1b and c). Moreover, similar results were also obtained in case of HCT116 xenograft tumours (Figure S2).

Additionally, as an alternative model for investigating the role of perivascular cells in tumour revascularization, HT‐29 tumour‐bearing mice were treated with PDGFRβ neutralizing antibodies to deplete vessel‐associated PDGFRβ^+^ perivascular cells (Figure 1d) (Cooke et al., 2012). The treatment of regorafenib and PDGFRβ antibodies significantly decreased the number of PDGFRβ^+^ tumour perivascular cells (Figure 1e and f). During the revascularization phase, tumours obtained following treatment with regorafenib and PDGFRβ antibodies were less revascularized and their CD31 density was remarkably lower than that of tumours obtained following treatment with regorafenib and IgG (Figure 1e and f). Collectively, depletion of PDGFRβ^+^ or α‐SMA^+^ perivascular cells impaired tumour revascularization after the discontinuance of AA‐TKI treatment.

### 3.2 Genetic ablation of perivascular cells attenuates off‐AA‐TKI‐triggered revascularization

**FIGURE 2:**
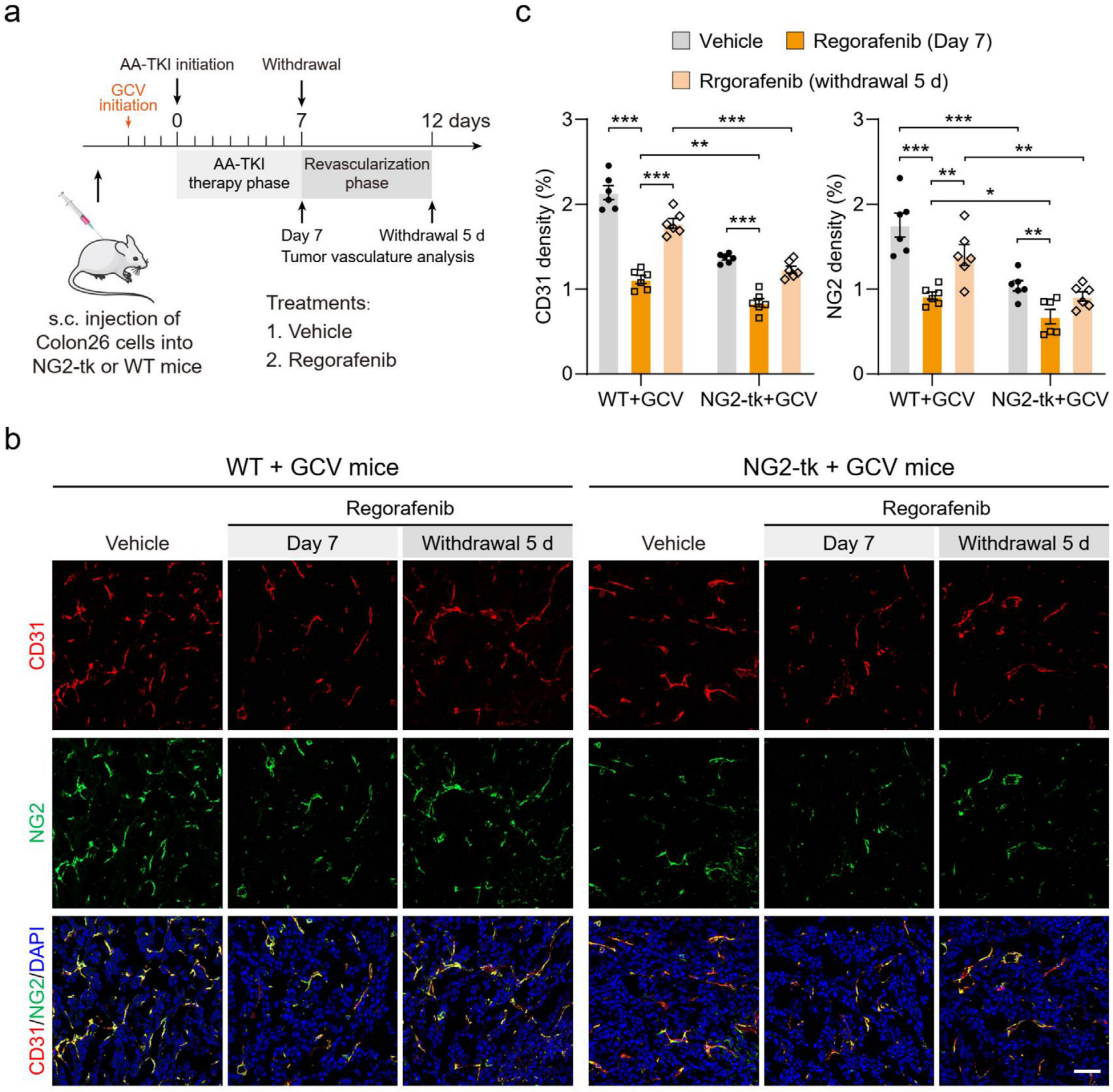
Reduced tumor revascularization after ablation of NG2^+^ perivascular cells. (a) Therapeutic schedule for treatment with vehicle and regorafenib. Subcutaneous inoculation of Colon26 cancer cells in NG2‐tk mice and WT littermates, with daily GCV (50 mg/kg) or saline injections when tumors reached approximately 200 mm^3^. Tumor vasculatures were analyzed at days 7 (Day 7) and 12 (withdrawal 5 d) after the indicated treatments. Withdrawal 5 d, 5 days after treatment withdrawal. (b) Representative images of CD31^+^ (red) endothelial cell, NG2^+^ (green) perivascular cell immunostaining, and DAPI (blue) for nuclear staining. Scale bars, 50 μm. (c) Quantification of microvessel density (CD31 density) and perivascular cell number (NG2 density) in Colon26 tumors (n = 6). Data are present as mean ± SEM. ^*^ *P* < 0.05, ^**^ *P* < 0.01, and ^***^ *P* < 0.001

To further elucidate the functional role of perivascular cells in tumour revascularization after cessation of regorafenib, transgenic mice that expressed viral thymidine kinase (tk) under control of the *Ng2* promoter (NG2‐tk mice) (Cooke et al., 2012) were used. Ganciclovir (GCV) treatment of NG2‐tk mice led to the selective depletion of proliferating NG2^+^ perivascular cells (Cooke et al., 2012). Then, mice CRC Colon26 tumours were established in GCV‐treated NG2‐tk mice (NG2‐tk+GCV) and their WT littermates (WT+GCV), followed by treatment with regorafenib (Figure 2a). Consistently, 7 days of regorafenib treatment significantly decreased microvessel density in Colon26 tumours from WT+GCV mice and Colon26 tumours showed dramatical revascularization after the withdrawal of regorafenib (Figure 2b and c). Expectedly, NG2^+^ perivascular cell ablation in Colon26 tumours were observed in NG2‐tk+GCV mice compared with those in WT+GCV mice (Figure 2b and c). In the revascularization phase (withdrawal 5 d), GCV‐induced NG2^+^ perivascular cell depletion led to deficient tumour revascularization and tumour microvessel density was much lower in NG2‐tk+GCV mice than that in WT+GCV mice (Figure 2b and c). These changes indicated that NG2^+^ perivascular cell ablation attenuated tumour vessel regrowth after cessation of regorafenib treatment. Taken together, all of the above data demonstrate that tumour perivascular cells are critically implicated in tumour revascularization after withdrawal of AA‐TKI therapy.

### 3.3 EPCs mediate perivascular cell‐induced revascularization after AA‐TKI withdrawal

**FIGURE 3:**
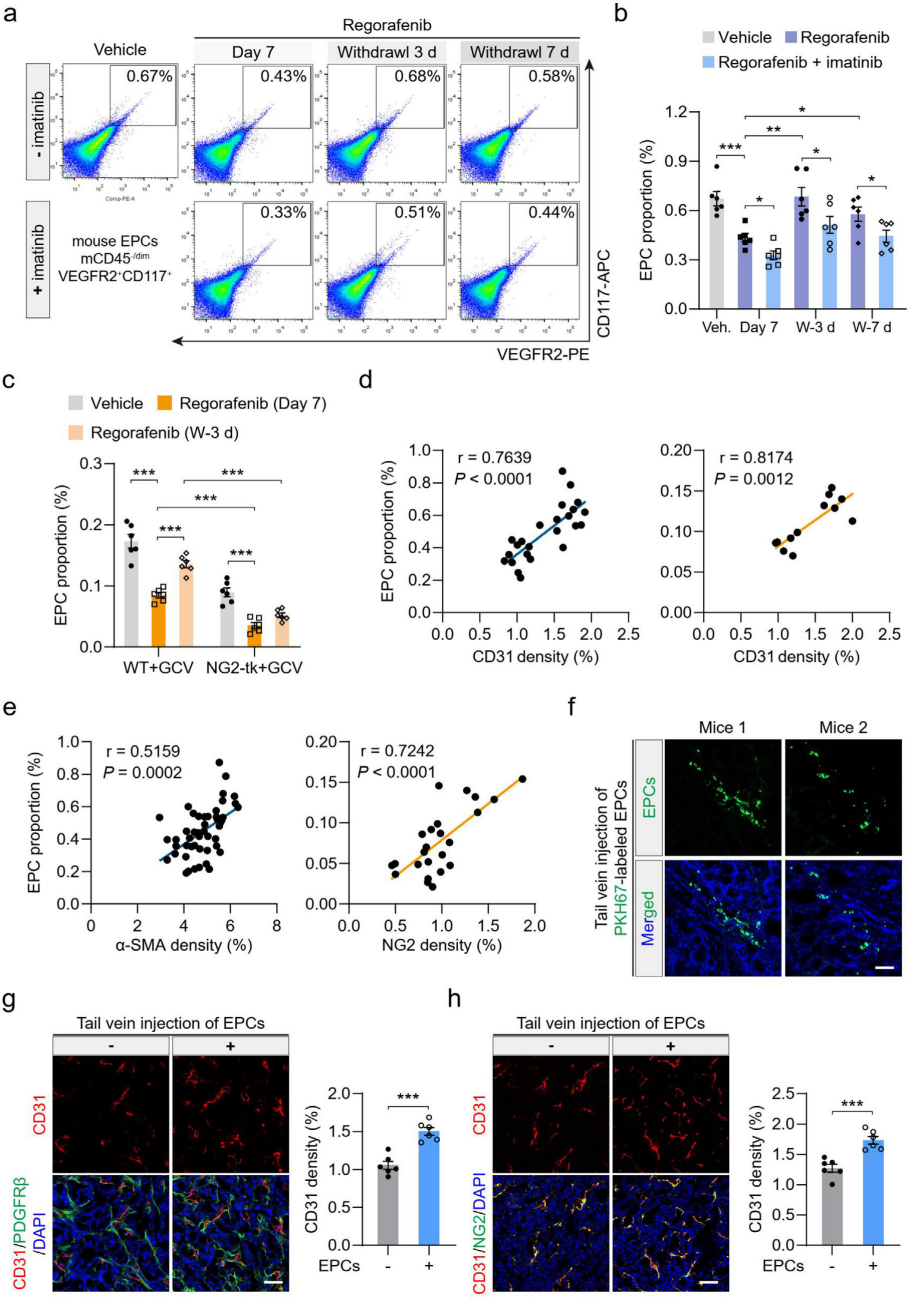
Tumor perivascular cells regulate EPC recruitment into tumors during the revascularization phase. (a and b) Quantification of the proportional changes of recruited EPCs in HT‐29 tumors (n = 6). Withdrawal 3/7 d, 3/7 days after AA‐TKI withdrawal. W‐3/7 d, 3/7 days after AA‐TKI withdrawal. (c) Quantification of the proportional changes of recruited EPCs in Colon26 tumors from NG2‐tk+GCV and WT+GCV mice after the indicated treatments (n = 6). (d) Pearson correlation analysis of EPC proportion and CD31 density in (d, left) HT‐29 tumors after treatment with AA‐TKIs for 7 days or withdrawal for 7 days; or in (d, right) Colon26 tumors from WT+GCV mice after treatment with regorafenib for 7 days or withdrawal for 5 days. (e) Pearson correlation analysis of (e, left) EPC proportion and perivascular cell number (α‐SMA density) in HT‐29 tumors after treatment with AA‐TKIs for 7 days or withdrawal for 7 days, or (e, right) EPC proportion and perivascular cell number (NG2 density) in Colon26 tumors from WT+GCV and NG2‐tk+GCV mice after treatment with regorafenib for 7 days or withdrawal for 5 days. (f) Representative images of PKH67‐labeled EPCs (green) recruited into HT‐29 tumors (mice 1) and Colon26 tumors (mice 2). DAPI (blue) was used for nuclear staining. (g) HT‐29 tumor‐bearing mice were treated with regorafenib + anti‐PDGFRβ for 7 days or (H) Colon26 tumor‐bearing NG2‐tk+GCV mice were treated with regorafenib for 7 days, followed by tail vein injection of 5 × 10^5^ EPCs every other day. Tumor microvessel density was determined after 5 days. Representative images of CD31^+^ (red) endothelial cell, PDGFRβ^+^ or NG2^+^ (green) perivascular cell immunostaining, and DAPI (blue) for nuclear staining. Quantification of CD31 density (n = 6). Data are present as mean ± SEM. Scale bars, 50 μm. ^*^ *P* < 0.05, ^**^ *P* < 0.01, and ^***^ *P* < 0.001

Increasing experimental evidence supports that EPC recruitment contributes to tumour neovascularization and that targeting EPCs is an attractive approach for antiangiogenic therapy (De La Puente et al., 2013; Shaked, 2006; Taylor et al., 2012). EPCs were characterized by several known markers, i.e., viable CD45^–/dim^/CD309 (VEGFR2)^+^/CD117 (c‐kit)^+^ cells (Ruan et al., 2013; Taylor et al., 2012). To investigate the role of perivascular cells in drug withdrawal‐induced EPC recruitment, HT‐29 tumour‐bearing mice were received treatment of regorafenib with or without imatinib. Then, flow cytometry (FCM) analysis was employed to determine the recruitment of EPC into tumours (Figure S3). The proportion of tumour‐recruited EPCs decreased by nearly 45% after treatment with regorafenib for 7 days and reduced by more than 55% after 7‐day treatment with regorafenib and imatinib (Figure 3a and b). During the revascularization phase (withdrawal 3 d or 7 d), the amount of tumour‐recruited EPCs was significantly smaller in tumours treated with combination therapy than that in tumours from mice treated with regorafenib solely (Figure 3a and b). Moreover, similar results were also recapitulated in HT‐29 tumour bearing mice treated with axitinib in the absence or presence of imatinib (Figure S1d). Consistently, in the revascularization phase, the number of EPCs recruited into Colon26 tumours was dramatically lower in NG2‐tk+GCV mice than that in WT+GCV mice (Figure 3c). Together, imatinib‐ or GCV‐induced perivascular cell ablation inhibits EPC recruitment in the revascularization phase.

We further investigated whether EPC recruitment was associated with drug withdrawal‐induced tumour revascularization. There was a positive correlation between EPC proportion and CD31 density in tumours (HT‐29 tumours, r = 0.7639, *P* < 0.0001; Colon26 tumours, r = 0.8174, *P* = 0.0012; Figure 3d). Additionally, EPC proportion was positively correlated with tumour perivascular cell number (α‐SMA or NG2 density) in tumours (α‐SMA density in HT‐29 tumours, r = 0.5159, *P* = 0.0002; NG2 density in Colon26 tumours, r = 0.7242, *P* < 0.0001; Figure 3e). These results indicated that EPCs participated in revascularization after AA‐TKI withdrawal and perivascular cell depletion inhibited EPC recruitment during the revascularization phase. Furthermore, whether increased EPC recruitment facilitated tumour revascularization was explored. EPCs were isolated from human umbilical cord blood and EPC identity was confirmed based on immunostaining for CD34, CD45, CD133, CXCR4, CD309 (VEGFR2), and UEA‐1, the uptake of ac‐LDL, and their morphological phenotype (Figure S4). PKH67‐labeled EPCs were intravenously injected into tumour‐bearing mice and they can be recruited to the tumour tissues after 24 h (Figure 3f). Remarkably, microvessel density was dramatically increased in HT‐29 (Figure 3g) and Colon26 tumours (Figure 3h) after the exogenous injection of EPCs. These data suggest that increased EPC recruitment enhanced tumour vessel regrowth. Collectively, EPC recruitment is regulated by tumour perivascular cells and crucially contributes to tumour revascularization after cessation of AA‐TKI therapy.

### 3.4 TPC‐EVs promote EPC recruitment and tumour revascularization

**FIGURE 4:**
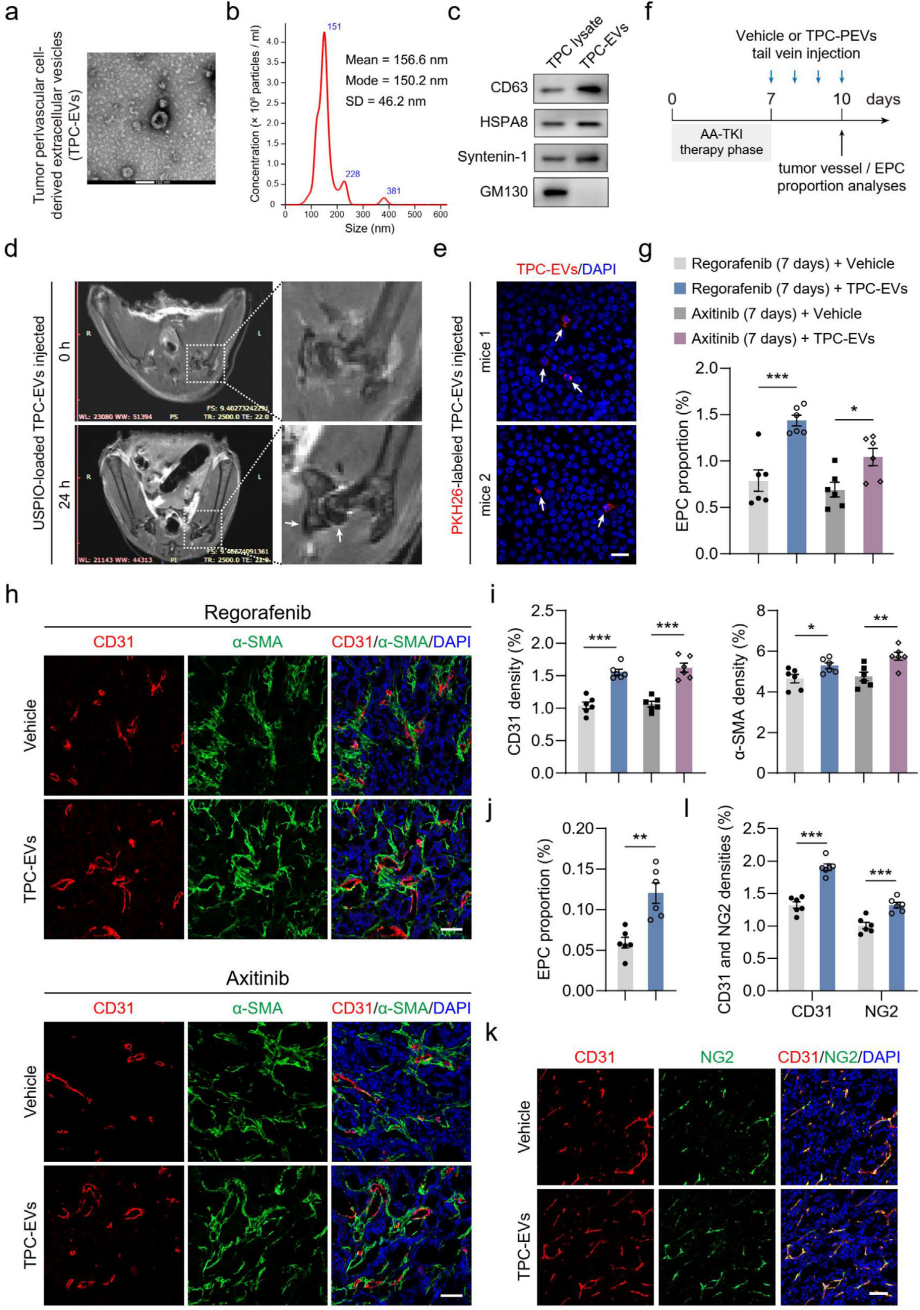
TPC‐EVs promote EPC recruitment into tumors to facilitate tumor revascularization. (a‐c) The characterization and quantification of TPC‐EVs by (a) TEM, (b) particle size, and (c) Western blotting analyses. (d) USPIO‐loaded TPC‐EVs (100 μg) were intravenously injected into mice and TPC‐EV incorporation into bone marrow was detected by magnetic resonance imaging analysis. White arrows indicated the accumulation of USPIO‐loaded TPC‐EVs. USPIO, ultrasmall superparamagnetic iron oxide nanoparticles. (e) PKH26‐labeled TPC‐EVs (100 μg) were intravenously injected into mice. Barrow cells were harvested after 24 h and then fixed and incubated with red blood cell lysis buffer. Representative images of incorporated TPC‐EVs (red) in marrow cells. Scale bar, 20 μm. (f) HT‐29 tumor‐bearing mice received the treatment of regorafenib or axitinib for 7 days, followed by tail vein injection with PBS (Vehicle) or TPC‐EVs (100 μg) for 4 days. Then, EPC recruitment and tumor vasculature were analyzed. (g) Quantification of the proportional changes of recruited EPCs in HT‐29 tumors (n = 6). (h) Representative images of CD31^+^ (red) endothelial cell, α‐SMA^+^ (green) perivascular cell immunostaining, and DAPI (blue) for nuclear staining. Scale bar, 50 μm. (i) Quantification of microvessel density (CD31 density) and perivascular cell number (α‐SMA density) in HT‐29 tumors (n = 6). Colon26 tumor‐bearing NG2‐tk+GCV mice were treated with regorafenib for 7 days, followed by tail vein injection of Vehicle or TPC‐EVs (100 μg) for 4 days. (j) Quantification of the proportional changes of recruited EPCs in Colon26 tumors (n = 6). (k) Representative images of CD31^+^ (red) endothelial cell, NG2^+^ (green) perivascular cell immunostaining, and DAPI (blue) for nuclear staining. Scale bar, 50 μm. (l) Quantification of microvessel density (CD31 density) and perivascular cell number (NG2 density) in Colon26 tumors (n = 6). Data are present as mean ± SEM. ^*^ *P* < 0.05, ^**^ *P* < 0.01, and ^***^ *P* < 0.001

EVs are important messengers for cell‐cell communications. Therefore, we further explored whether tumour perivascular cells promoted EPC recruitment for revascularization via an EV‐mediated mechanism. Primary TPCs were isolated from clinical surgical specimens and cultured in vitro (Figure S5a), which were identified by TEM, immunostaining, and FCM analyses (Figure S5b‐d). TPC‐EVs were harvested, characterized, and quantified. The TEM image showed TPC‐EVs with a typical cup‐shaped morphology (Figure 4a). NTA results showed that the mean diameter, mode diameter, and concentration of TPC‐EVs were 156.6 ± 46.5 nm, 150.2 nm, and 2.24 × 10^8^ particles/ml, respectively (Figure 4b). Western blotting analysis verified that TPC‐EVs were positive for CD63, HSPA8, and Syntenin‐1, but negative for GM130, a Golgi apparatus marker (Figure 4c). Since the bone marrow is the primary origin of EPCs, the accumulation of TPC‐EVs in the bone marrow was determined as previously described (Busato et al., 2016; Hoshino et al., 2015). Magnetic resonance imaging (MRI) analysis showed that ultrasmall superparamagnetic iron oxide nanoparticles (USPIO)‐loaded TPC‐EVs were incorporated into the bone marrow (Figure 4d). Immunostaining further validated that bone marrow cells took up PKH26‐labeled TPC‐EVs (Figure 4e).

Next, TPC‐EVs were intravenously injected into tumour‐bearing mice treated with regorafenib or axitinib for 7 days (Figure 4f). FCM analysis revealed that a nearly 2‐fold increase in tumour EPC recruitment was seen in mice after 4 days of TPC‐EV treatment (Figure 4g). TPC‐EV treatment significantly promoted revascularization and increased microvessel density in HT‐29 tumours (Figure 4h and i) and HCT116 tumours (Figure S6). Consequently, the proliferation index of tumour cells was also increased by nearly 1.5‐fold following treatment with TPC‐EVs (Figure S7). Moreover, TPC‐EV treatment significantly increased the number of recruited EPCs in Colon26 tumours (Figure 4j) and promoted tumour revascularization (Figure 4k and l). Together, these data indicates that TPC‐EVs promote tumour revascularization via EPC recruitment.

### 3.5 TPC‐EVs promote EPC vasculogenesis

**FIGURE 5:**
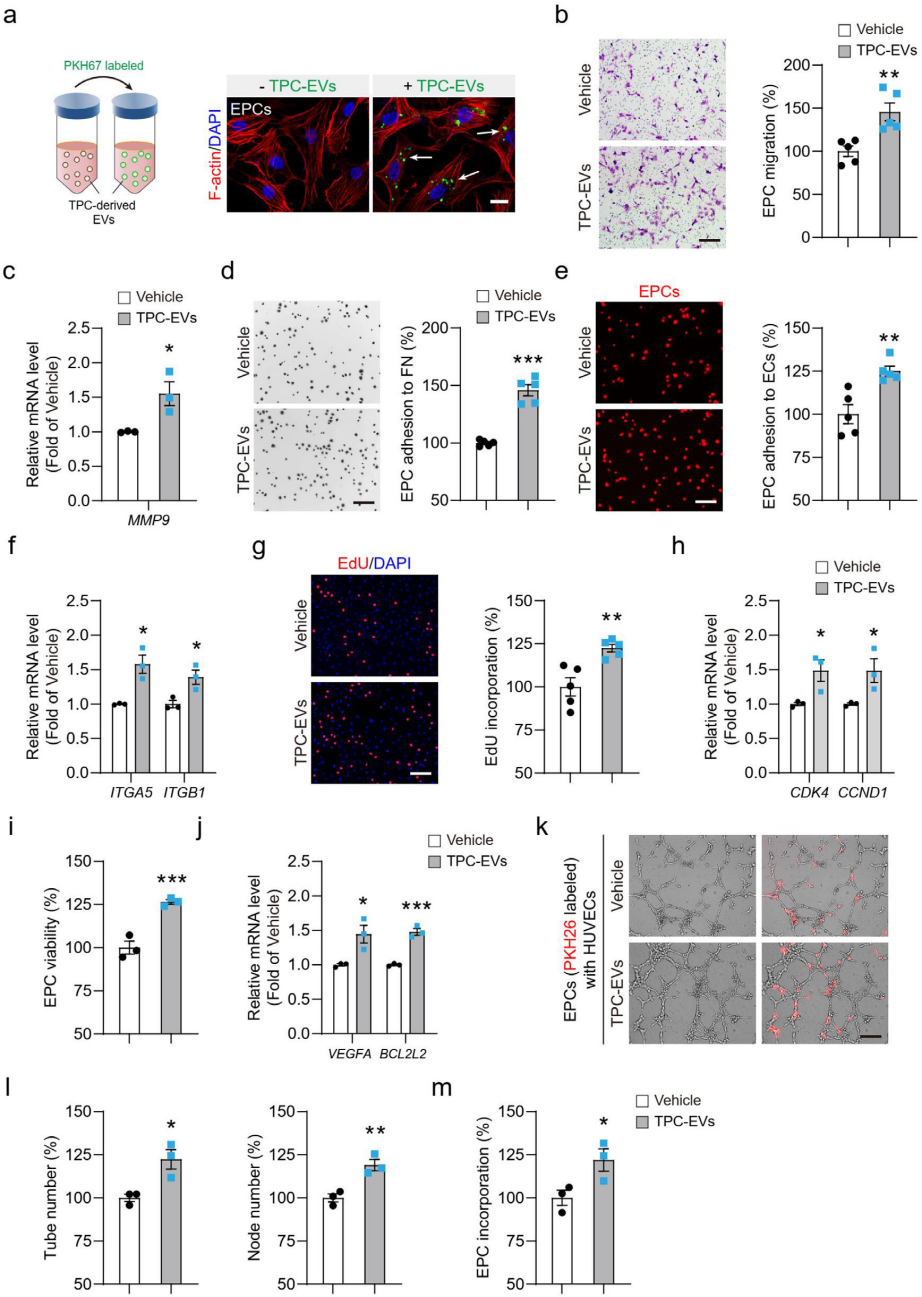
TPC‐EVs promote EPC vasculogenesis in vitro. (a) Uptake of TPC‐EVs in EPCs. TPC‐EVs were labeled with PKH67 and then incubated with EPCs for 2 h. Rhodamine phalloidin‐TRITC was used for cytoskeletal protein (F‐actin) staining and DAPI was used for nuclear staining. White arrows indicated TPC‐EV uptake by EPCs. Scale bar, 20 μm. (b) Quantification of the number of migrated cells (n = 5). (c) Quantitative assessment of *MMP9* mRNA level in EPCs after TPC‐EV treatment (n = 3). (d and e) Representative images of EPC adhered to (d) fibronectin and (e) HUVECs monolayers, and quantification of the number of EPCs adhered to fibronectin or HUVECs (n = 5). FN, fibronectin; ECs, HUVECs. (f) RT‐PCR assay for *ITGA5* and *ITGB1* mRNA levels in EPCs after TPC‐EV treatment (n = 3). (g) Representative images of EdU^+^ EPCs and quantification of EdU incorporation (n = 5). (h) Quantification of *CDK4* and *CCND1* mRNA levels in EPCs (n = 3). (i) EPC viability was measured by MTT assay (n = 3). (j) Quantitative assessment of *VEGFA* and *BCL2L2* mRNA expression in EPCs (n = 3). (k) Representative images of EPC/EC co‐cultured tubule structures. Quantification of (l) the tube and node number of co‐culture tubule structures and (m) the number of EPC incorporated into endothelial tubes (n = 3). Data are presented as mean ± SEM. Scar bar, 200 μm. ^*^ *P* < 0.05, ^**^ *P* < 0.01, and ^***^ *P* < 0.001 versus Vehicle groups

Next, we attempted to explore the mechanisms underlying the action of TPC‐EVs in EPC‐mediated tumour revascularization. EPCs were used for in vitro studies and exhibited a significant uptake capacity of TPC‐EVs (Figure 5a). The ability of TPC‐EVs to stimulate EPC chemotaxis, a critical step in EPC recruitment (Maeng et al., 2009), was first tested. Consistent with the *in vivo* study (Figure 4g and j), a 1.5‐fold increase of EPC migration was observed after TPC‐EV treatment (Figure 5b). TPC‐EV stimulation also significantly increased the *MMP9* mRNA level in EPCs (Figure 5c). Progenitor cell adhesion to the extracellular matrix and endothelial cells was also crucial for their homing to the damaged tissues[34]. TPC‐EVs markedly increased the number of EPC adhered to fibronectin (Figure 5d) and human umbilical vein endothelial cell (HUVEC) monolayers (Figure 5e). Moreover, a previous study reported that α5‐ and β1‐integrins were important for the adhesion and migration of EPCs (Kim et al., 2015). TPC‐EV treatment also significantly increased the mRNA levels of *ITGA5* and *ITGB1* in EPCs (Figure 5f). Besides, TPC‐EV treatment significantly enhanced EPC proliferation (Figure 5g) and increased the mRNA levels of *CDK4* and *CCND1* in EPCs (Figure 5h). TPC‐EVs promoted EPC survival under serum deprivation condition and upregulated *VEGFA* and *BCL2L2* in EPCs (Figure 5i and j). Moreover, the effect of TPC‐EVs on EPC vasculogenesis was further examined by a Matrigel tube formation assay and EPCs and HUVECs were co‐seeded onto Matrigel. TPC‐EVs potently stimulated vascular structure formations (Figure 5k) and significantly increased the tube or node number of lumen structures (Figure 5l). TPC‐EVs also markedly promoted EPC incorporation into the HUVEC/EPC tubule structures (Figure 5k and m). Taken together, our results show that TPC‐EVs promote EPC vasculogenic properties in vitro.

### 3.6 Extracellular vesicle‐Gas6 is critical for EPC vasculogenic properties and tumour revascularization

**FIGURE 6:**
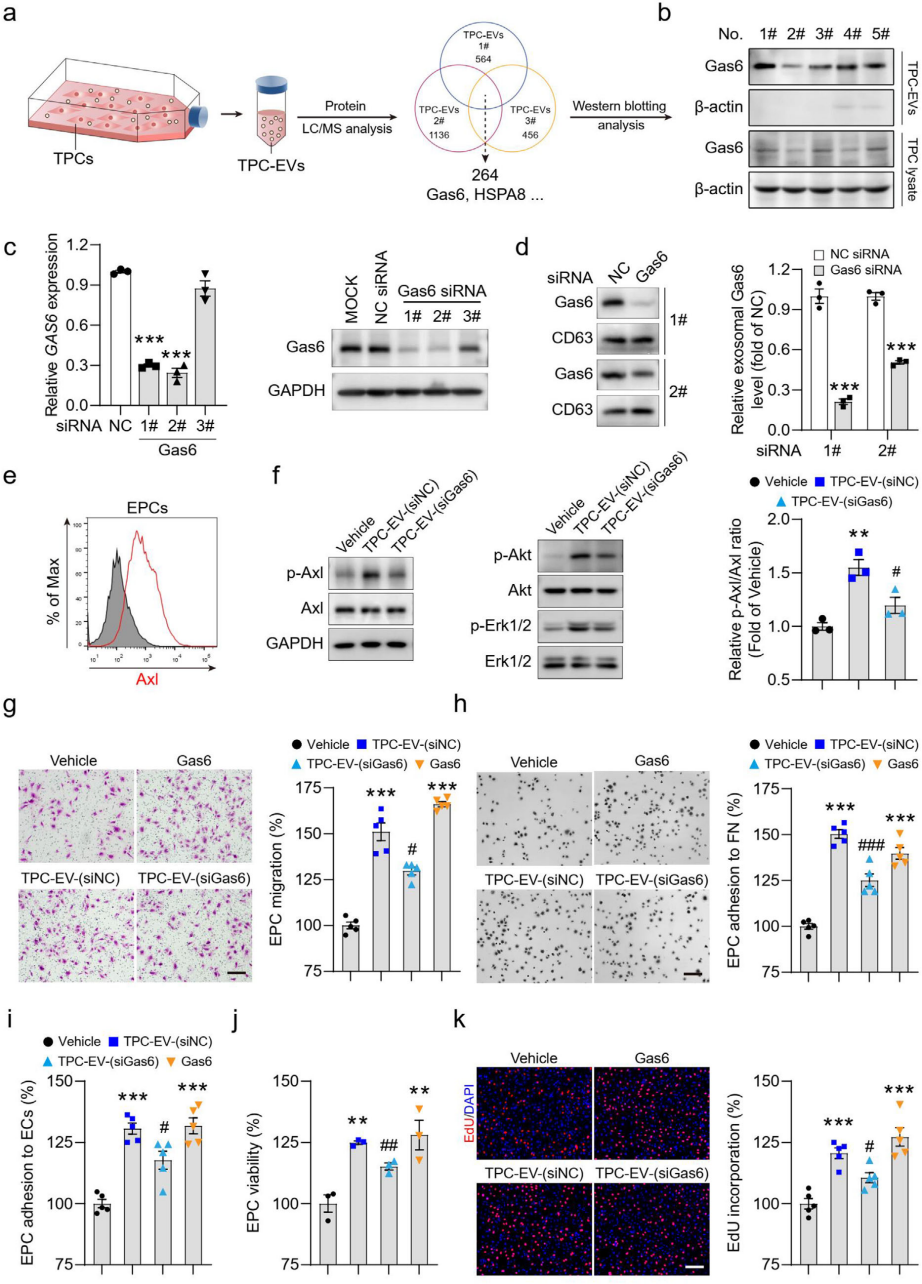
TPC‐EV‐induced EPC vasculogenic properties are regulated by Gas6 in vitro. (a) LC‐MS/MS (nanoLC‐QE) proteomic analysis for TPC‐EVs. (b) The protein level of Gas6 in tumor perivascular cell lysates and TPC‐EVs were determined by Western blotting assay. No., number order. (c) Gas6 mRNA and protein levels in tumor perivascular cells after transfection with Gas6 siRNA were analyzed by RT‐PCR and Western blotting assays. Quantification of Gas6 mRNA expression in tumor perivascular cells after transfection with Gas6 siRNAs (n = 3). ^***^ *P* < 0.001 versus the NC siRNA‐transfected group by one‐way ANOVA and Tukey's multiple comparison test. (d) EVs were harvested from tumor perivascular cells transfected with Gas6 siRNA and EV‐Gas6 protein levels were determined by Western blotting analysis. CD63 served as a loading control. Quantification of Gas6 expression in TPC‐EVs (n = 3). ^***^ *P* < 0.001 versus the NC siRNA‐transfected groups by unpaired two‐tailed *t*‐test. (e) Flow cytometry analysis of Axl expression in EPCs. (f) The phosphorylated and total forms of Axl, Akt and Erk1/2 in EPCs after the indicated treatments were determined. Representative blots and quantification of the ratio of p‐Axl/Axl (n = 3). TPC‐EV‐(siNC), EVs derived from TPCs transfected with NC siRNA. TPC‐EV‐(siGas6), EVs derived from TPCs transfected with Gas6 siRNA. (g) Representative images and quantification of the number of migrated EPCs after the indicated treatments (n = 5). (h) Representative images and quantification of the number of EPC adhered to FN. FN, fibronectin. (i) Quantification of the number of EPC adhesion to HUVECs (n = 5). ECs, HUVECs. (j) Quantitative assessment of EPC viability after the indicated treatments (n = 3). (k) EPCs were treated with TPC‐EV‐(siNC), TPC‐EV‐(siGas6), and Gas6 (200 ng/ml) for 48 h, and then EPC proliferation was examined. Quantification of EdU incorporation in EPCs (n = 5). Data are presented as the mean ± SEM. Scar bar, 200 μm. ^**^ *P* < 0.01, and ^***^ *P* < 0.001 versus the Vehicle groups; ^#^ *P* < 0.05, ^##^ *P* < 0.01, and ^###^ *P* < 0.001 versus the TPC‐EV‐(siNC)‐treated groups

Protein composition is a key feature of EVs (Kalluri, 2016). In this study, the protein components of TPC‐EVs were identified by LC‐MS/MS (nanoLC‐QE) analysis (Figure 6a). There were 264 common proteins in three independent preparations of EVs from different CRC perivascular cells (Supplementary Table 4). Since the Gas6/Axl pathway regulated endothelial survival (O'donnell et al., 1999) and angiogenesis (Lei et al., 2016), EV‐derived Gas6 (EV‐Gas6) was selected for further investigation (Figure 6b). To figure out whether TPC‐EV‐induced EPC angiogenic function was mediated by EV‐Gas6, Gas6 was depleted in tumour perivascular cells by transfecting with Gas6‐specific siRNAs (Figure 6c) and Gas6‐deficient TPC‐EVs (TPC‐EV‐(siGas6)) were generated (Figure 6d). FCM analysis validated that EPCs were positive for Axl (Figure 6e). TPC‐EV‐(siNC) induced a 1.5‐fold increase in the phosphorylated level of Axl receptor and activated its downstream effectors Akt and Erk1/2 in EPCs, which was significantly reduced in TPC‐EV‐(siGas6)‐treated EPCs (Figure 6f). Compared to TPC‐EV‐(siNC), TPC‐EV‐(siGas6) showed significantly decreased abilities to promote EPC migration (Figure 6g) and to increase the number of EPC adhered to fibronectin (Figure 6h) or HUVEC monolayers (Figure 6i). Moreover, Gas6 depletion attenuated the ability of TPC‐EVs to promote EPC survival (Figure 6j) and proliferation (Figure 6k). These data indicate that TPC‐EV‐induced EPC vasculogenic properties may be regulated by EV‐Gas6 *in vitro*.

**FIGURE 7:**
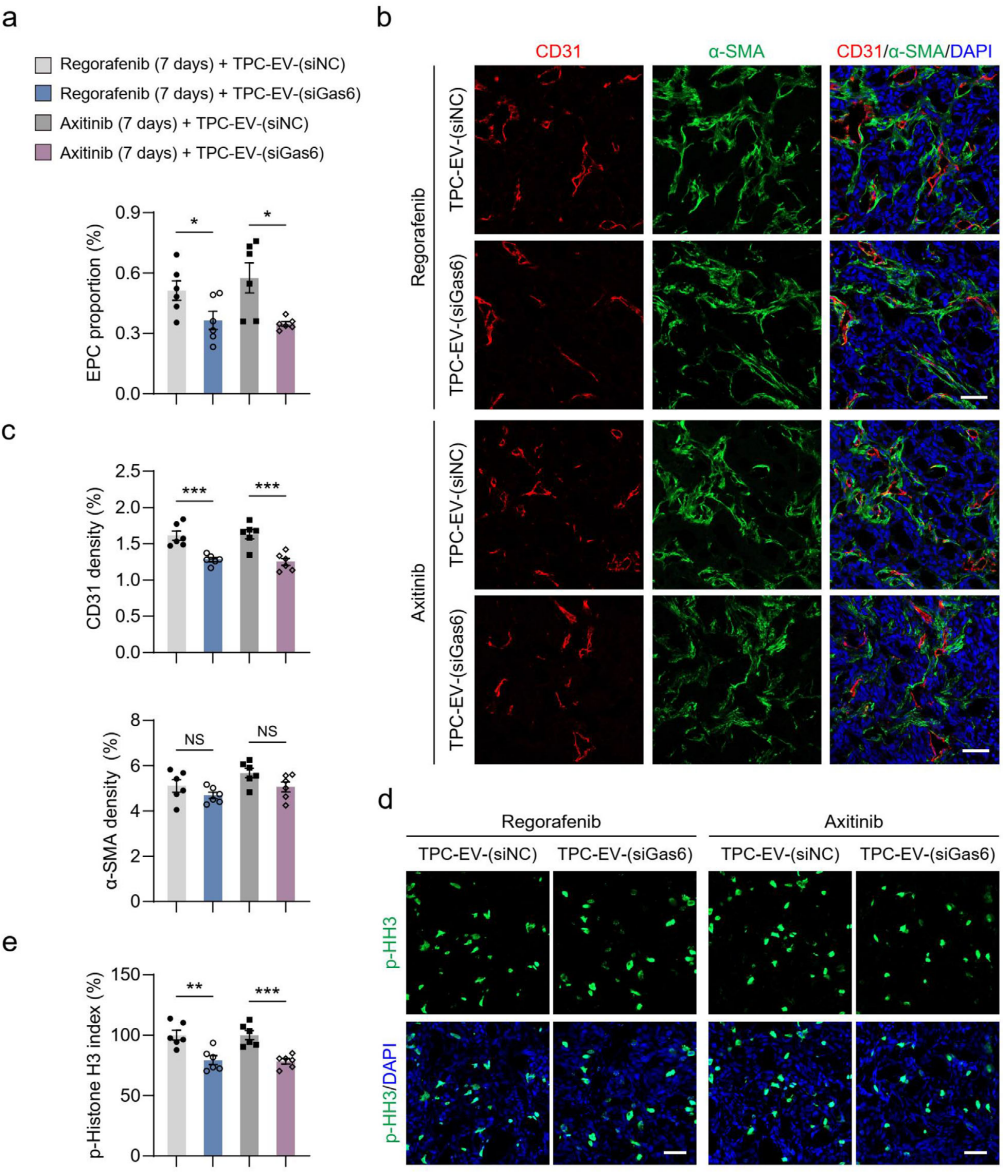
TPC‐EV‐mediated EPC recruitment and tumor revascularization are regulated by Gas6 *in vivo*. (a) Quantification of the proportional changes of recruited EPCs in HT‐29 tumors (n = 6). (b) Representative images of CD31^+^ (red) endothelial cell, α‐SMA^+^ (green) perivascular cell immunostaining, and DAPI (blue) for nuclear staining. (c) Quantification of microvessel density (CD31 density) and perivascular cell number (α‐SMA density) in HT‐29 tumors (n = 6). NS, no significance. (d) Representative images of cells with p‐Histone H3 positive staining (green) and DAPI (blue) for nuclear staining. (e) Quantification of cell proliferation (p‐Histone H3 index) in HT‐29 tumors (n = 6). Data are presented as the mean ± SEM. Scale bars, 50 μm. ^*^ *P* < 0.05, ^**^ *P* < 0.01, and ^***^ *P* < 0.001

Next, whether TPC‐EV‐induced EPC recruitment and tumour revascularization was regulated by TPC‐EV‐Gas6 was determined. Tumour‐bearing mice that received 7 days of AA‐TKI treatment were treated with TPC‐EV‐(siNC) or TPC‐EV‐(siGas6). Compared to TPC‐EV‐(siNC), TPC‐EV‐(siGas6) showed decreased ability to promote EPC recruitment into tumours (Figure 7a). Tumour microvessel density was significantly lower in the TPC‐EV‐(siGas6)‐treated groups than that in the TPC‐EV‐(siNC)‐treated groups (Figure 7b and c). The proliferative index of cancer cells was dramatically decreased after treatment with TPC‐EV‐(siGas6) when compared with TPC‐EV‐(siNC) (Figure 7d and e). Collectively, these data suggest that TPC‐EV‐derived Gas6 is involved in promoting EPC recruitment for tumour revascularization by after AA‐TKI therapy.

### 3.7 Blockade of the EPC‐Axl pathway suppresses TPC‐EV‐induced EPC vasculogenic properties

**FIGURE 8:**
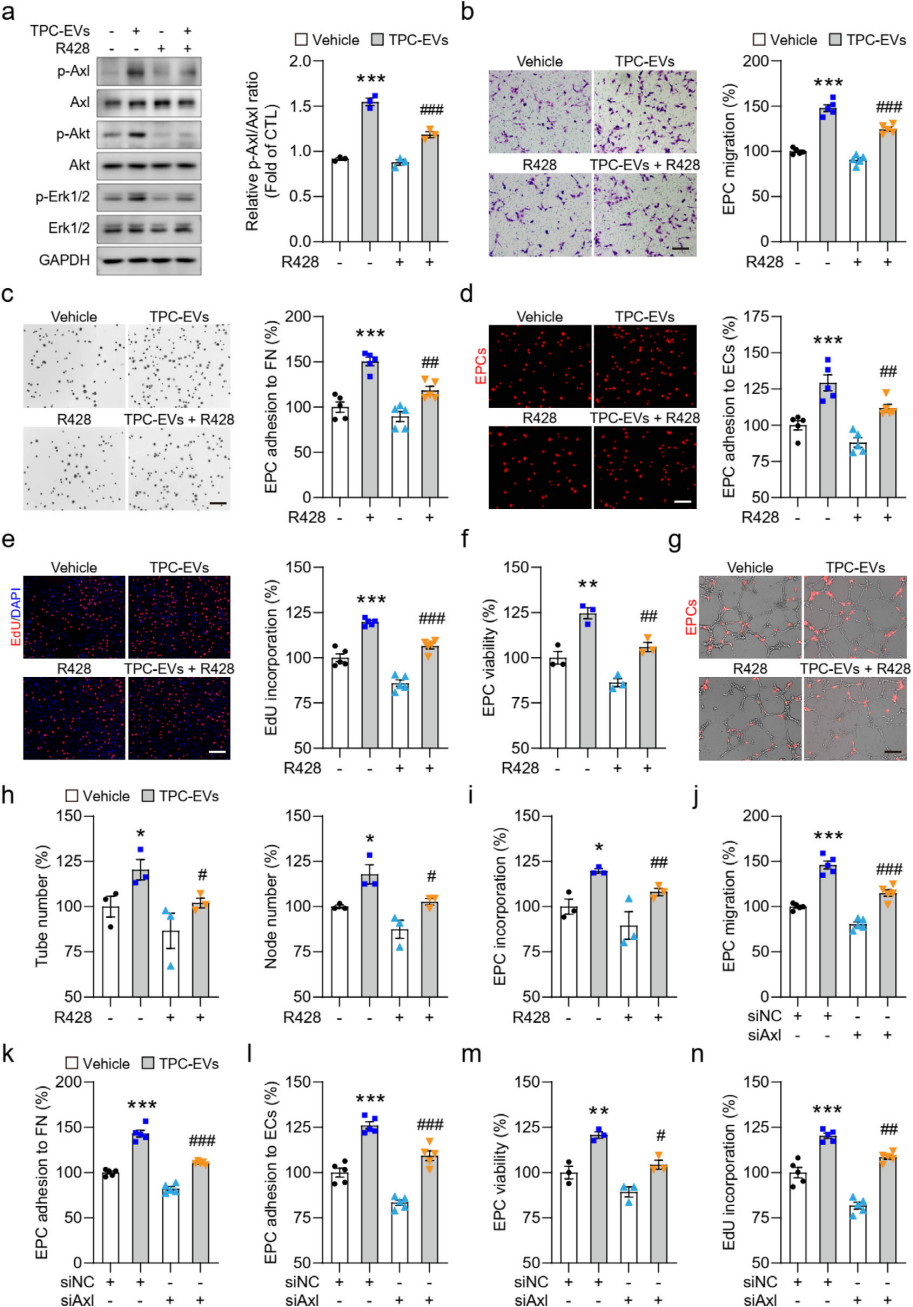
TPC‐EV‐induced EPC vasculogenic properties are dependent on Axl pathway activation. (a) Representative blots and quantification of the ratio of p‐Axl/Axl in EPCs after the indicated treatments (n = 3). (b) Transwell migration assay of EPC migration capacity after treatment with TPC‐EVs and R428. Quantification assessment of migrated EPCs (n = 5). (c and d) Representative images of EPC adhesion to (c) fibronectin or (d) HUVEC monolayers. Quantification of EPC adhered to (c) fibronectin and (d) HUVEC monolayers (n = 5). FN, fibronectin; ECs, HUVECs. (e and f) EPCs were incubated with TPC‐EVs with or without R428 (0.25 μM), and then (e) cell proliferation and (f) viability were determined at 48 and 24 h, respectively. (g) Representative images of EPC/HUVEC tubule structures and quantification of the number of (H) tube, node and (I) incorporated EPCs of EPC/HUVEC tubule structures (n = 3). (J) Silencing Axl inhibited TPC‐EV‐mediated EPC migration (n = 5). (k and l) Axl siRNA attenuated TPC‐EV‐induced EPC adhered to fibronectin and HUVEC monolayers. Quantification of EPC adhered to (k) fibronectin and (l) HUVEC monolayers (n = 5). FN, fibronectin; ECs, HUVECs. (m and n) Quantification of EdU incorporation (n = 5) and viability (n = 3) in EPCs after TPC‐EV treatment. siNC, NC siRNA. siAxl, Axl siRNA. Scar bar, 200 μm. Data are presented as mean ± SEM, n = 3 or 5. ^*^ *P* < 0.05, ^**^ *P* < 0.01, and ^***^ *P* < 0.001 versus the Vehicle groups. ^#^ *P* < 0.05, ^##^ *P* < 0.01, and ^###^ *P* < 0.001 versus the TPC‐EV‐treated groups

We next examined whether TPC‐EV‐induced EPC vasculogenic properties were dependent on Axl pathway activation. The Axl specific inhibitor R428 (Holland et al., 2010) significantly inhibited the TPC‐EV‐induced phosphorylation of Axl, Akt, and Erk1/2 in EPCs (Figure 8a). R428 treatment significantly inhibited TPC‐EV‐induced EPC migration (Figure 8b) and reduced the number of EPC adhered to fibronectin (Figure 8c) and HUVEC monolayers (Figure 8d). Additionally, the proliferation rate and viability in EPCs were decreased after R428 plus TPC‐EV treatment compared to those in EPCs treated with TPC‐EV alone (Figure 8e and f). TPC‐EV‐induced EPC/HUVEC tubule formation was also dramatically suppressed by R428 treatment (Figure 8g and h). The number of EPC incorporation into the EPC/HUVEC tubule networks was significantly decreased in the TPC‐EVs plus R428‐treated groups (Figure 8g and i). Furthermore, the mRNA levels of angiogenesis‐related genes in EPCs were increased by TPC‐EV treatment, which were suppressed by R428 treatment (Figure S8).

The role of Axl pathway activation in TPC‐EV‐induced EPC vasculogenic properties was further confirmed by Axl silencing. Both mRNA and protein levels of Axl were markedly downregulated in EPCs transfected with Axl siRNAs (Figure S9a and b). Similar to R428 treatment, the knockdown of Axl blocked TPC‐EV‐induced phosphorylation of Axl, Akt, and Erk1/2 in EPCs (Figure S9c). Silencing of Axl impaired TPC‐EV‐induced EPC migration (Figure 8j) and adhesion capacities (Figure 8k and l). Furthermore, we also observed a significant decrease in the ability of TPC‐EVs to promote the proliferation (Figure 8m) and survival (Figure 8n) of Axl‐silenced EPCs, compared to the case for EPCs transfected with NC siRNA. Together, TPC‐EV‐induced EPC vasculogenic properties are dependent on Axl pathway activation in vitro.

### 3.8 Inhibition of the Axl signalling suppresses EPC recruitment and tumour revascularization

**FIGURE 9:**
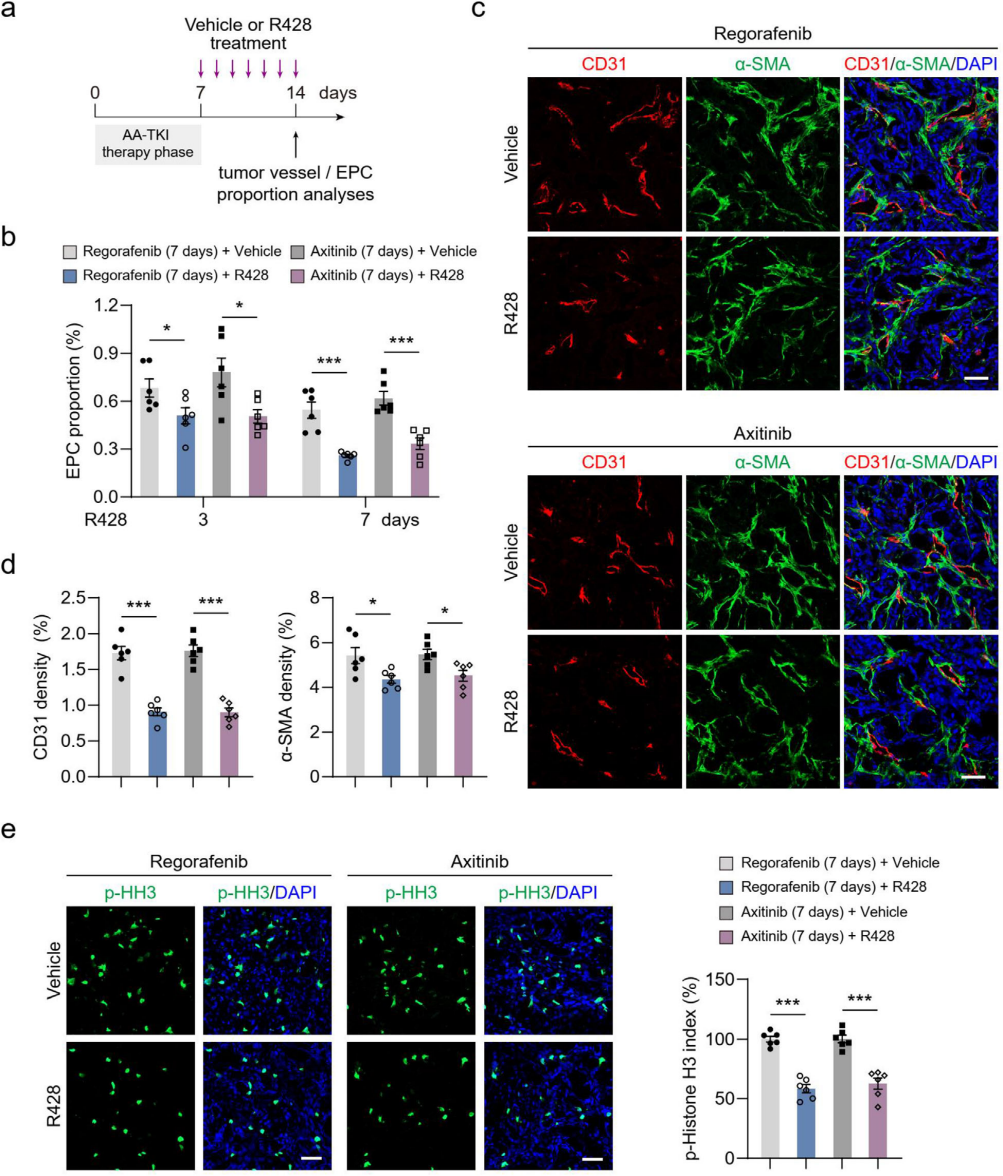
Inhibition of the Axl pathway impairs EPC recruitment and suppresses tumor revascularization after AA‐TKI treatment. (a) Therapeutic schedule for R428 administration in HT‐29 tumor‐bearing mice after regorafenib or axitinib treatment. (b) Quantification of the proportional changes of recruited EPCs in HT‐29 tumors (n = 6). (c) Representative images of CD31^+^ (red) endothelial cell, α‐SMA^+^ (green) perivascular cell immunostaining, and DAPI (blue) for nuclear staining. (d) Quantification of microvessel density (CD31 density) and perivascular cell number (α‐SMA density) in HT‐29 tumors (n = 6). (e) Representative images of cells with p‐Histone H3 positive staining (green) and DAPI (blue) for nuclear staining. Quantification of cell proliferation (p‐Histone H3 index) in HT‐29 tumors (n = 6). Data are presented as mean ± SEM. Scale bars, 50 μm. ^*^ *P* < 0.05, ^**^ *P* < 0.01, and ^***^ *P* < 0.001

To further confirm the role of Axl pathway in EPC recruitment and tumour revascularization in vivo, HT‐29 tumour‐bearing mice received regorafenib or axitinib treatment for 7 days, followed by intragastric administration with R428 (Figure 9a). FCM analysis revealed that R428 treatment drastically decreased the proportion of EPCs recruited into tumours (Figure 9b). Notably, R428 treatment also impaired tumour revascularization and led to significant decreases in microvessel density (Figure 9c and f, and Figure S10) and tumour cell proliferation (Figure 9e). Collectively, blockage of the Axl pathway inhibits EPC recruitment and attenuates tumour revascularization after AA‐TKI therapy cessation.

### 3.9 Regorafenib and R428 dual treatment prolongs overall survival and reduces tumour burden in mice with metastatic CRC

**FIGURE 10:**
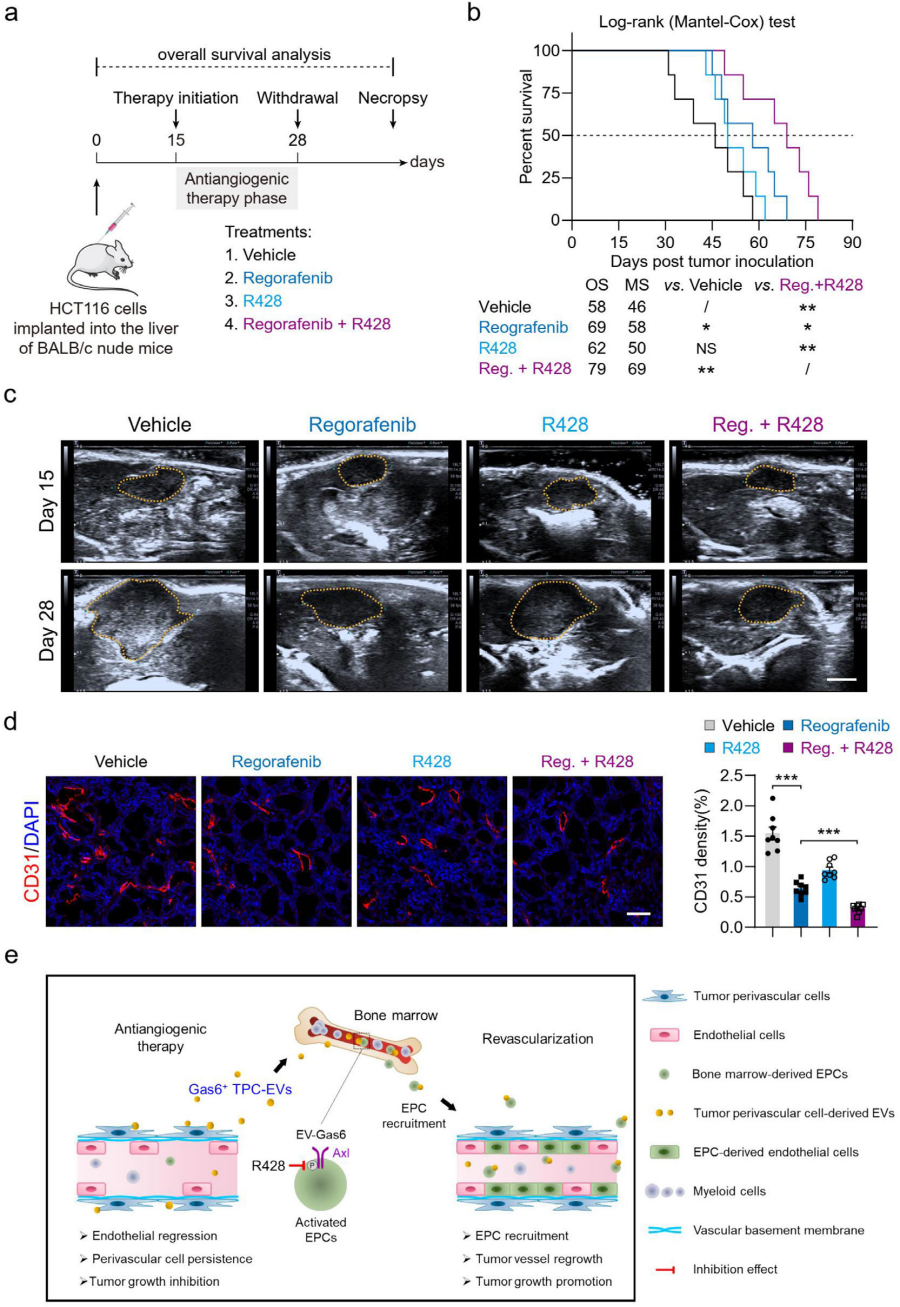
Regorafenib plus R428 therapy prolongs survival and decreases the tumor burden in mice with colorectal cancer liver metastases. (a) Therapeutic schedule for continuous treatment with vehicle, regorafenib, R428, and regorafenib + R428 in mice bearing HCT116 liver metastases. (b) Kaplan‐Meier estimates of overall survival for mice with HCT116 hepatic tumors after the indicated treatment (n = 7). OS, overall survival. MS, median survival. Reg., regorafenib. NS, no significance. ^*^ *P* < 0.05 and ^**^ *P* < 0.01 versus indicated groups by Log‐rank (Mantel‐Cox) test. (c) Representative ultrasound images of HCT116 hepatic tumors after the indicated treatments. Mice bearing HCT116 hepatic tumors were treated as indicated and imaged at days 15 and 28 after tumor implantation. Scale bar, 5 mm. (d) Representative images of CD31^+^ (red) endothelial cell immunostaining and DAPI (blue) for nuclear staining. Scale bar, 50 μm. Quantification of microvessel density (CD31 density) in HCT116 hepatic tumors (n = 8). Data are presented as mean ± SEM. ^***^ *P* < 0.001. (e) Diagram depicting the mechanism by which Gas6^+^ TPC‐EVs activates the Axl pathway and consequently promotes EPC recruitment for rapid tumor revascularization after withdrawal of AA‐TKI therapy

The above results demonstrated that TPC‐EV‐derived Gas6 activated the Axl signalling pathway in EPCs and promoted EPC recruitment, which limited the therapeutic efficacy of AA‐TKIs. Therefore, we examined whether the inhibition of the Axl pathway in EPCs could improve the anti‐cancer efficacy of regorafenib in mice with colorectal cancer liver metastases. Colorectal cancer HCT116 liver metastases models were established, and tumour angiogenesis, tumour burden, and the OS of tumour‐bearing mice after the indicated treatments were analyzed (Figure 10a). Compared to vehicle groups (OS, 58 days), regorafenib significantly prolonged the survival time of HCT116 hepatic tumour‐bearing mice (OS, 69 days). The OS was further prolonged in mice treated with regorafenib plus R428 (OS, 79 days) (Figure 10b). Further, treatment of regorafenib and R428 exerted a stronger inhibition in tumour growth and microvessel density than regorafenib monotherapy (Figure 10c, d). Collectively, these results suggest that R428 blocks tumour perivascular cell EV‐Gas6‐mediated activation of the Axl signalling pathway in EPCs, enhances the therapeutic efficacy of regorafenib, and increases the survival time of mice with metastatic colorectal cancer.

## 4 DISCUSSION

CRC is the third most diagnosed cancer in both men and women and is globally the second cause of cancer‐related death worldwide (Bray et al., 2018). AA‐TKIs, such as regorafenib, fruquintinib, and axitinib, are commonly used as monotherapies for treating advanced or metastatic CRC. However, approximately 25% of patients experience AA‐TKI treatment discontinuation due of adverse events, economic reasons, or drug resistance (Motzer et al., 2013). The cessation of anti‐angiogenic therapy often results in tumour revascularization, thus accelerating tumour regrowth and metastatic spread (Ebos et al., 2009; Haemmerle et al., 2016; Mancuso et al., 2006). This phenomenon, also called flare‐up, has been widely reported in both preclinical and clinical practices (Desar et al., 2009; Powles et al., 2013; Yang et al., 2016). Here, our data demonstrate that perivascular cells mediate tumour revascularization after withdrawal of regorafenib or axitinib treatment. Mechanistic investigations show that TPC‐EVs promote EPC recruitment for tumour revascularization after AA‐TKI cessation. Blockage of the EV‐Gas6/Axl pathway significantly suppresses EPC recruitment and tumour revascularization, which could be a potential therapy to enhance the efficacy of AA‐TKI against CRC (Figure 10e).

Perivascular cells are implicated in tumour angiogenesis (Ferland‐Mccollough et al., 2017; Raza et al., 2010) and they may represent a novel target for anti‐angiogenic therapy (Chen et al., 2017; Ferland‐Mccollough et al., 2017; Ruan et al., 2013). However, no specific maker has been defined to identify and characterize tumour perivascular cells thus far, which makes it challenging to isolate perivascular cells from tumour tissues. Currently, the majority of *in vitro* researches on perivascular cells are performed on human brain vascular pericytes (HBVPs) and HBVPs are co‐cultured with cancer cells or transfected with FAPα to mimic tumour perivascular cells (Chen et al., 2017; Franco et al., 2011). However, the features of educated or genetically modified HBVPs are substantially distinct from tumour perivascular cells; thus, the establishment of a suitable isolation method for tumour perivascular cell is essential for research associated with tumour perivascular cells. In the present study, we successfully obtain tumour perivascular cells from human CRC vessels using a novel approach. Tumour vessels are isolated from surgical specimens and perivascular cells are purified by the conditional culturing with perivascular cell medium. To our knowledge, our group is the first to obtain CRC perivascular cells and our work provides an evidential and valuable example for future studies of tumour perivascular cells.

While there are several preliminary studies on perivascular cell‐derived EVs, the contents of tumour perivascular cell‐derived EVs and their role in tumour angiogenesis and vascular functions remain elusive. In our study, proteomic analysis reveals 264 common proteins in three independent preparations of EVs derived from different tumour perivascular cells and the majority of proteins are ribosomal proteins, membrane proteins, and cytoskeletal proteins, such as collagens and laminins. These results indicate that the protein component and function of TPC‐EVs may be diverse and multifunctional. In our study, Gas6 is present in TPC‐EVs and instigated EPC recruitment for tumour revascularization via activating the Axl pathway. TPC‐EV treatment increased both CD31 and α‐SMA densities in tumour tissues when compared to the vehicle groups; however, the differences of α‐SMA densities in TPC‐EV‐(siNC)‐ and TPC‐EV‐(siGas6)‐treated groups were not statistically significant, which indicated that there might be several EV proteins in TPC‐EV‐(siGas6) that could regulate the proliferation and/or recruitment of α‐SMA‐positive cells. In addition to the EV‐Gas6/Axl pathway, various signalling pathways are also associated with the regulation of EPC angiogenic properties (De La Puente et al., 2013), and thus there also may be a significant and notable response in EPCs without the EV‐Gas6/Axl pathway. Indeed, in addition to ribosomal and cytoskeletal proteins that might show less effect on EPC angiogenic phenotypes and revascularization, several membrane proteins also are present in TPC‐EVs. Among them, Annexin A2 that has been reported to regulate the angiogenesis activities of endothelial cells (Maji et al., 2017), is present in TPC‐EVs and whether it could regulate EPC vasculogenic properties needs to be studied in the future.

The mechanisms of EV uptake and cargo transmission into recipient cells, particularly in EV protein‐mediated signal transduction cascade in acceptor cells, are complex and largely unknown (Mathieu et al., 2019). On one hand, EVs could be internalized by recipient cells via endocytosis or pinocytosis and then recycled back to the extracellular space. For instance, EV‐derived angiopoietin‐2 from hepatocellular carcinoma was delivered into recipient HUVECs via endocytosis and it can be recycled to induce angiogenesis by activating the Akt/eNOS and Akt/β‐catenin axes (Xie et al., 2020). On the other, EVs can transfer information into recipient cells by acting at the cell surface. Soluble E‐cadherin was reportedly localized on the surface of EVs from ovarian cancer cells and it can bind to and heterodimerize with VE‐cadherin on endothelial cells, which activated the β‐catenin and NF‐κB pathways to promote angiogenesis (Tang et al., 2018). VEGFA was found at either the inner or outer surface of EVs from glioblastoma stem‐like cells and EV‐VEGFA still retained its pro‐angiogenic activity (Treps et al., 2017). These studies indicated that EV‐cytokines could be transferred into recipient cells via endocytosis‐associated recycling or directly interact with membrane proteins on recipient cells to trigger signal transduction cascades (Mathieu et al., 2019). In our study, we found that tumour perivascular cell EV‐Gas6 retained its pro‐angiogenic activity via activating the Axl pathway in EPCs; however, the mechanisms of underlying the interaction of EV‐Gas6 with the Axl receptor and EV‐Gas6‐mediated Axl receptor activation in EPCs are still unknown. Here, we can hypothesize that EV‐Gas6 could be delivered into EPCs via endocytosis, followed by its recycling to activate the Axl pathway, or that Gas6 might be localized on the EV surface and interact with, and activate the Axl receptor in EPCs when EVs fuse with EPC membrane. In addition to proteins, miRNAs and lncRNAs are the major components of EVs (Kalluri, 2016). EV‐derived miRNA and lncRNA derived from tumour cells are confirmed to regulate tumour angiogenesis and vascular functions (Yu & Wang, 2018; Zeng et al., 2018). Thus, we should also investigate the role of EV‐derived miRNAs or lncRNAs from tumour perivascular cells in the regulation of EPC‐mediated tumour revascularization. In addition to EVs (Liu et al., 2019; Yuan et al., 2019), chemokines and growth factors secreted from perivascular cells were also crucial to angiogenesis and vascular functions (Mcguire et al., 2011; Uemura et al., 2018). Therefore, further research is needed to elucidate the effect of tumour perivascular cell‐derived cytokines on EPC recruitment and tumour revascularization.

Bone marrow‐derived EPCs, a subtype of cells with stemness and high proliferation capacity, can differentiate into mature endothelial cells to regulate neovascularization under physiologic and pathologic conditions (De La Puente et al., 2013; Shi et al., 1998). In addition to acute vascular injury models, such as models of ischemic stroke and hind‐limb ischemia (Hayakawa et al., 2012; Kim et al., 2015), EPCs also play an important role in tumour vasculatures. EPCs are recruited into ischemic or hypoxic tumours predominantly in response to chemokines (e.g., CXCL12, CCL2, CCL5) and growth factors (e.g., VEGF, bFGF), which interact with their respective receptors (e.g., CXCR4, CCR2, CCR5, VEGFR2) on the surface of EPCs (De La Puente et al., 2013). These chemokines and growth factors are mainly secreted by tumour cells and stromal cells, such as tumour endothelial cells and cancer‐associated fibroblasts (De La Puente et al., 2013; Orimo et al., 2005; Spring et al., 2005). For example, the chemokines CCL2 and CCL5 produced by the neovessels of late‐stage tumours, attracted CCR2^+^ and CCR5^+^ EPCs into tumours, which led to tumour neovascularization (Spring et al., 2005). The CXCL10/CXCR3 axis can induce the recruitment and differentiation of EPCs and promote the formation of neovessels, which facilitated liver tumour growth after transplantation (Ling et al., 2014). Herein, we found that tumour perivascular cell EV‐Gas6 facilitated EPC recruitment into tumours and promoted tumour vessel regrowth after AA‐TKI treatments. Collectively, our data demonstrated that the EV‐Gas6/Axl axis is a novel signal mode in the regulation of EPC vasculogenic properties and recruitment into tumours, which provided a more in‐depth understanding of the role of tumour perivascular cell‐mediated EPC recruitment in tumour revascularization.

In conclusion, our results demonstrate that persistent perivascular cells confer tumour revascularization after withdrawal of regorafenib or axitinib treatment via an EV‐mediated manner. TPC‐EV‐derived Gas6 promotes EPC recruitment into tumours for revascularization by activating the Axl pathway. Our study also suggests that targeting either TPC‐EV‐derived Gas6 or the Axl pathway in EPCs can be a promising therapeutic strategy for enhancing the therapeutic efficacy of AA‐TKIs in CRC.

## CONFLICTS OF INTEREST

The authors declare no competing interests.

## AUTHOR CONTRIBUTIONS

Dongmei Zhang, Wencai Ye and Yihai Cao designed and supervised the experiments, and revised the manuscript. Tongzheng Liu, Patrick Ming‐Kuen Tang, An Hong and Yunlong Yang provided critical reading and revision of the manuscript. Maohua Huang and Minfeng Chen wrote the manuscript and analyzed the data. Maohua Huang, Minfeng Chen, Ming Qi, and Geni Ye performed animal experiments. Changzheng Shi and Xukai Mo performed magnetic resonance imaging analysis and analyzed the data. Luyu Zhao performed ultrasound image assay and analyzed the data. Maohua Huang, Geni Ye, Xiaobo Li, and Jiayan Zhang performed flow cytometry analysis and analyzed the data. Maohua Huang, Minfeng Chen, Ming Qi, Yong Li, Weijin Lu, and Jincheng Zhong performed immunofluorescence assay and image acquisition. Maohua Huang, Minfeng Chen, and Geni Ye performed cell line studies and Western blotting assay. Jinghua Pan and Yiran Zhang collected human CRC tissues. Jinrong Lin collected human umbilical cord blood samples. Jinghua Pan and Liangping Luo reviewed the pathological section and assessed the preclinical and clinical samples.

## Supporting information

Supporting information.

Supporting information.
