# Supplementary material for: Perivascular cell‐derived extracellular vesicles stimulate colorectal cancer revascularization after withdrawal of antiangiogenic drugs
Source: J Extracell Vesicles. 2021 May 21;10(7):e12096. doi: 10.1002/jev2.12096 (PMC8138700; doi:10.1002/jev2.12096)
Supplement: Supplementary file 1 — Supporting information. [file JEV2-10-e12096-s001.docx]

**Supplementary Materials for**

**Perivascular cell-derived extracellular vesicles stimulate colorectal cancer revascularization after withdrawal of antiangiogenic drugs**

Maohua Huang, Minfeng Chen, Ming Qi, Geni Ye, Jinghua Pan, Changzheng Shi, Yunlong Yang, Luyu Zhao, Xukai Mo, Yiran Zhang, Yong Li, Jincheng Zhong, Weijin Lu, Xiaobo Li, Jiayan Zhang, Jinrong Lin, Liangping Luo, Tongzheng Liu, Patrick Ming-Kuen Tang, An Hong, Yihai Cao^*^, Wencai Ye^*^, Dongmei Zhang^*^

*Corresponding authors. E-mail: dmzhang701@jnu.edu.cn (D.M.Z); chywc@aliyun.com (W.C.Y); Yihai.cao@ki.se (Y.H.C).

**Supplementary figures and figure legends**


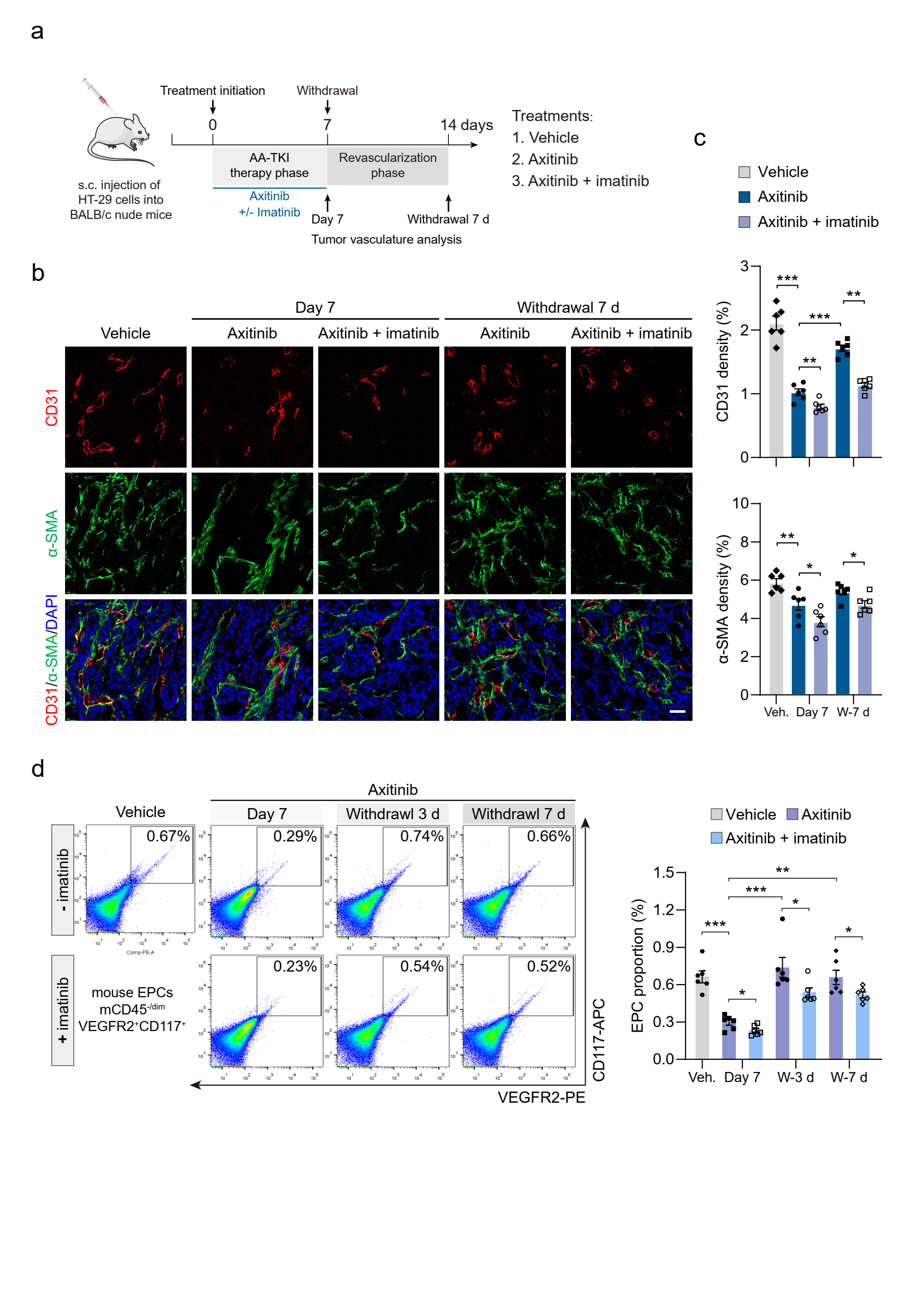


**Supplementary Figure 1.** **Tumor perivascular cells mediate tumor revascularization and EPC recruitment after withdrawal of axitinib.** (**a**) Therapeutic schedule for treatment with vehicle, axitinib, and axitinib + imatinib. Tumor vasculatures were analyzed at days 7 (Day 7) and 14 (withdrawal 7 d) after the indicated treatments. Withdrawal 7 d, 7 days after AA-TKI withdrawal. (**b**) Representative images of CD31^+^ (red) endothelial cell, α-SMA^+^ (green) perivascular cell immunostaining, and DAPI (blue) for nuclear staining. Scale bars, 50 μm. (**c**) Quantification of microvessel density (CD31 density) and perivascular cell number (α-SMA density) in HT-29 tumors (n = 6). (**d**) Quantification of the proportional changes of recruited EPCs in HT-29 tumors (n = 6). Veh., Vehicle. W-3/7 d, 3/7 days after AA-TKI withdrawal. Data are present as mean ± SEM. ^*^*P* < 0.05, ^**^*P* < 0.01, and ^***^*P* < 0.001.


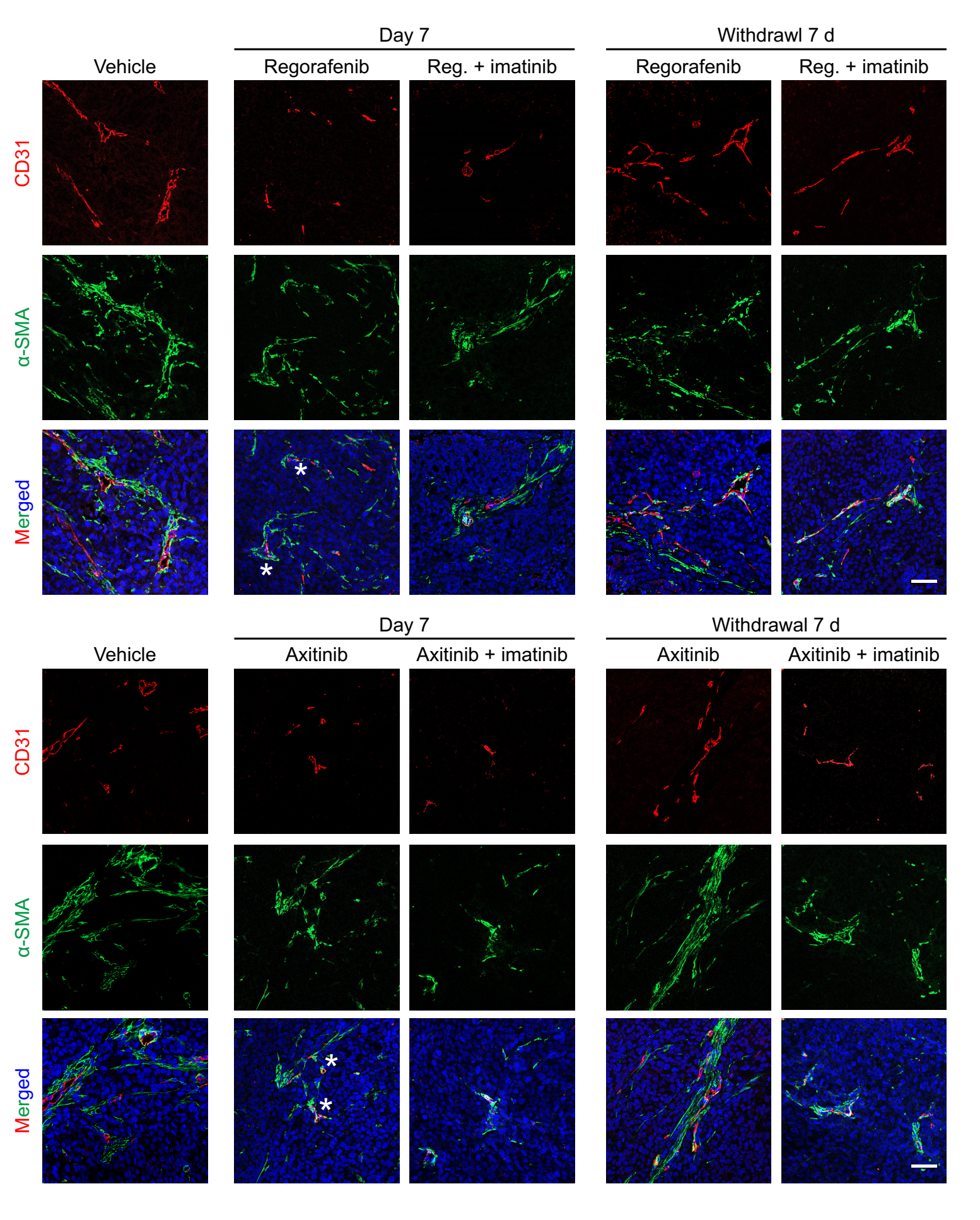


**Supplementary Figure 2. Tumor perivascular cells mediate HCT116 tumor revascularization after AA-TKI treatment.** Mice bearing HCT116 tumor were treated with Vehicle, regorafenib, regorafenib + imatinib, axitinib, and axitinib + imatinib, and tumor vasculature was analyzed at days 7 (Day 7) and 14 (withdrawal 7 d) after the indicated treatments. Reg., Regorafenib. Withdrawal 7 d, 7 days after AA-TKI withdrawal. Representative images of CD31^+^ (red) endothelial cell, α-SMA^+^ (green) perivascular cell immunostaining, and DAPI (blue) for nuclear staining in tumors after the indicated treatments. Scale bar, 50 μm. White asterisks (*) indicate surviving tumor vessels.


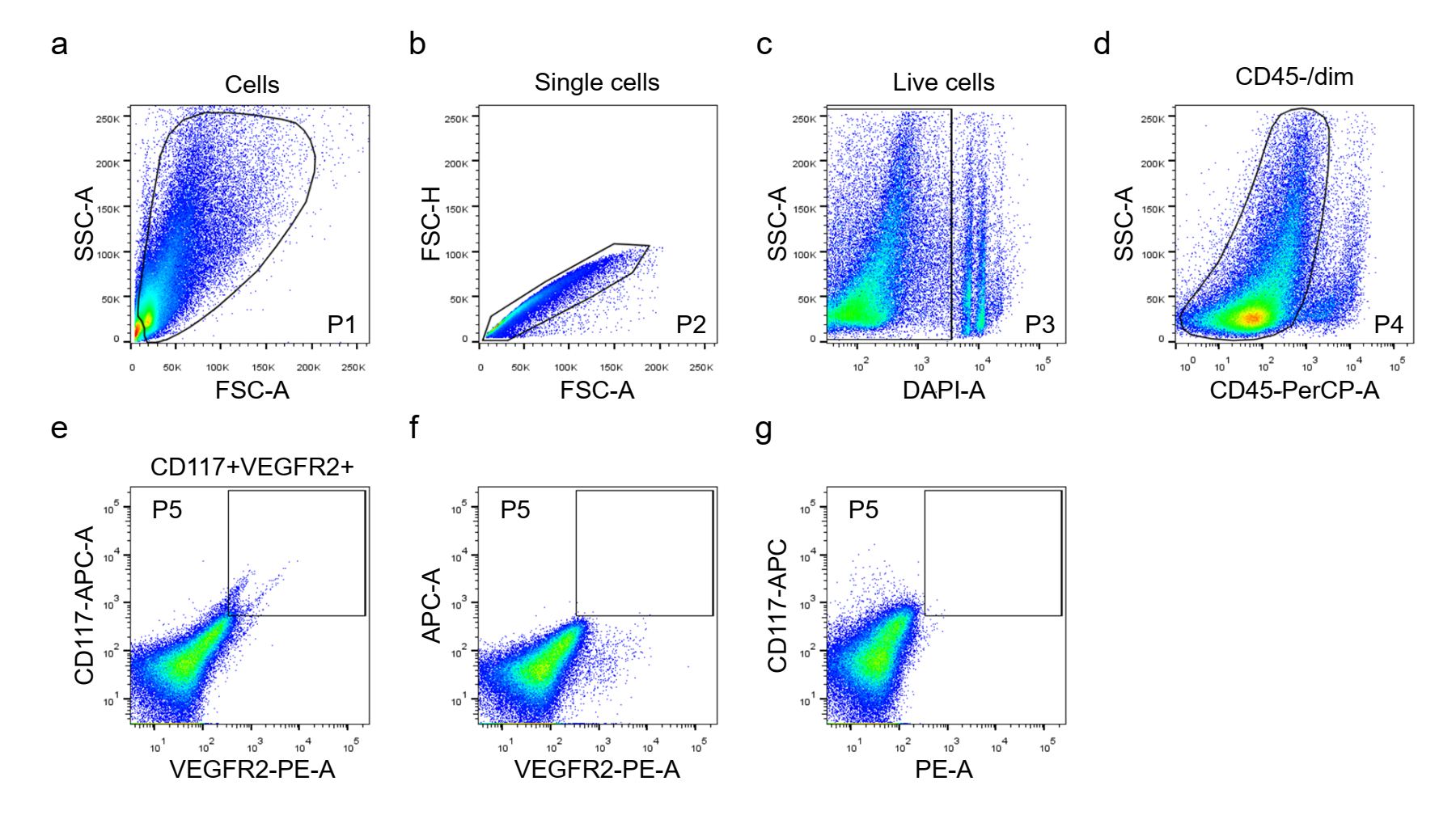


**Supplementary Figure 3.** **Representative flow cytometry analysis of EPCs recruited into tumors.** (**a**) Large initial morphological gate to exclude red blood cells, non-viable/apoptotic debris (about 60% of acquired events are shown). (**b** and **c**) Overlapped and adhesive cells and non-viable/apoptotic cells are gated out to select DAPI-negative viable events. (**d** and **e**) Bi-exponential representations for the analysis of the successive gates used to identify EPCs as CD45^-/dim^/CD117^+^/ VEGFR2^+^ events (shown in gate P5). (**f** and **g**) Analysis of the FMO controls for APC (**f**) and PE (**g**) using a gating strategy identical to that used for the test tube (events gated in P4 are shown).


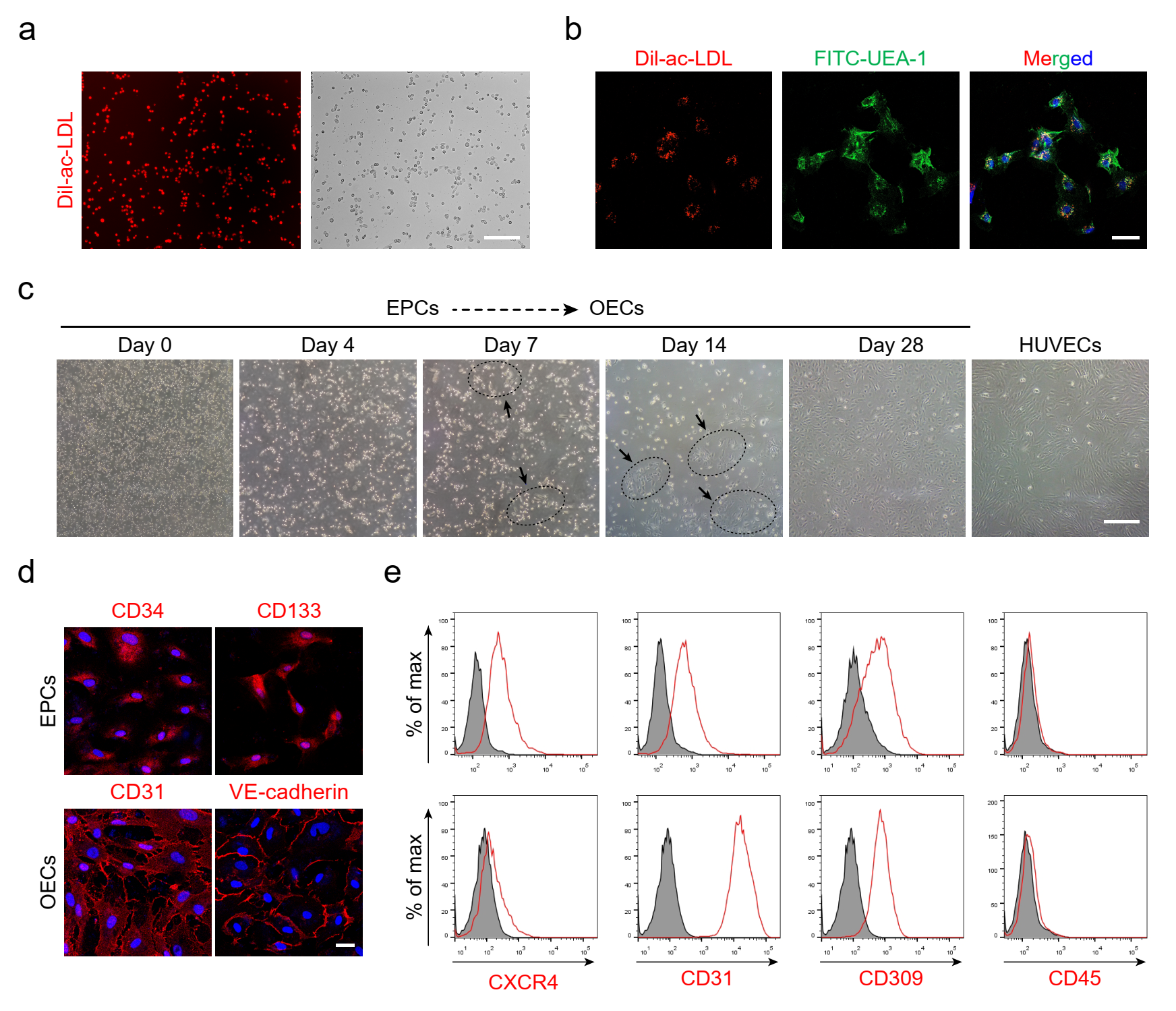


**Supplementary Figure 4.** **Isolation, culture, and identification of human cord blood-derived EPCs.** (**a-d**) Representative phase contrast and fluorescence microscopy fields of EPCs. (**a)** Early EPCs after culture for 5 days and immunofluorescence staining revealed Dil-ac-LDL uptake in EPCs. (**b**) 7-day EPCs were double stained with Dil-ac-LDL and FITC-UEA-1. DAPI (blue) was used for nuclear staining. (**c**) The morphological changes and differentiation of EPCs. Typical colonies of outgrowth endothelial cells (OECs, sometimes also called late EPCs) were visible at 14 days and extended EPC culture led to confluent monolayers of cells with a mature EC morphology. Black arrows indicate EPCs colonies. Scale bar, 200 μm. (**d**) Immunofluorescence staining of CD34, CD133 (red) characterized early EPCs (EPCs) and increased fluorescence intensity of CD31 and VE-cadherin (red) staining identified late EPCs (OECs). DAPI staining indicated cell nuclei. (**e**) Flow cytometry analysis of surface markers of EPCs and OECs. EPCs marker: CXCR4. Hematopoietic cells marker, CD45. Endothelial cells markers, CD31 and CD309 (VEGFR2). Scale bar, 200 μm (**a** and **c**); 50 μm (**b**); 20 μm (**d**).


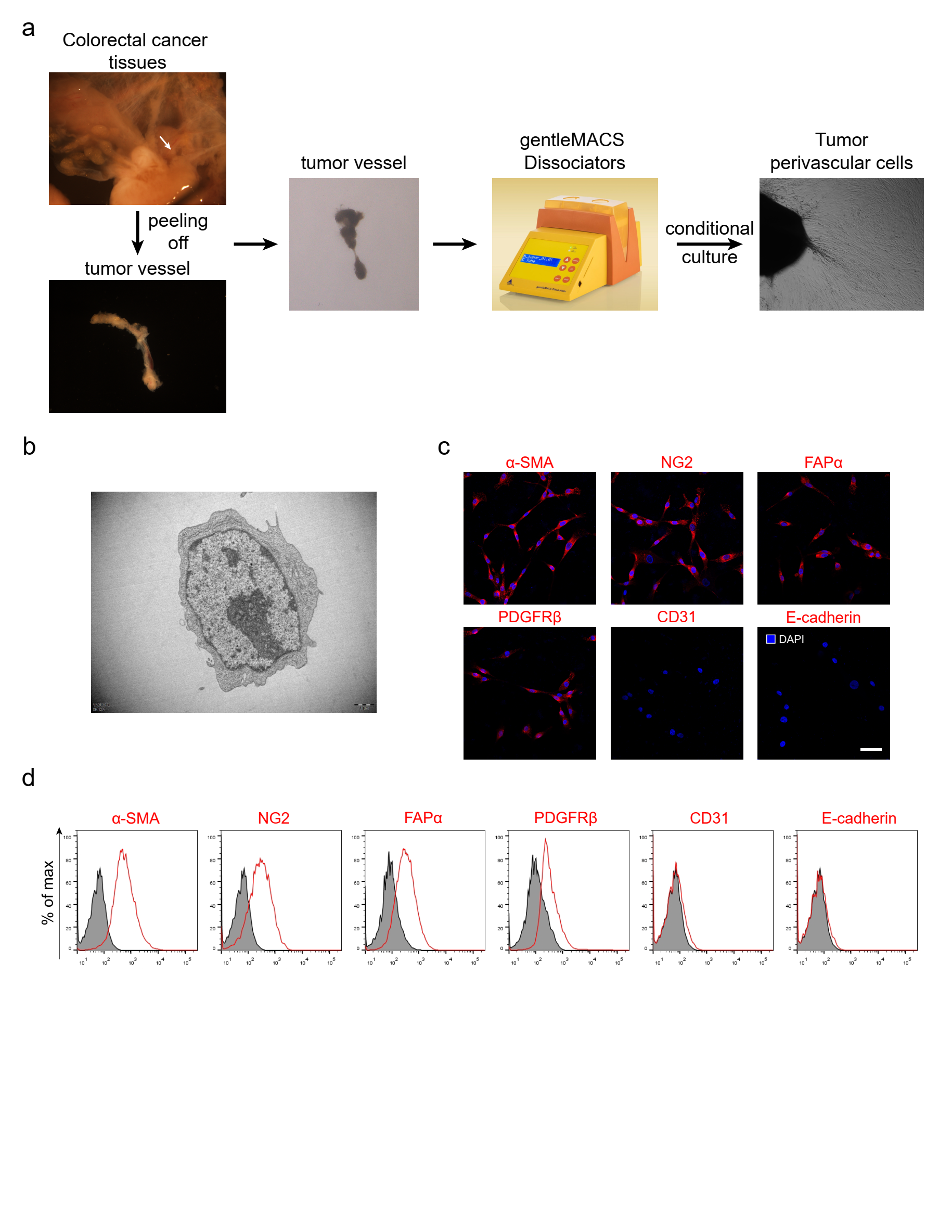


**Supplementary Figure 5.** **Isolation, culture, and identification of perivascular cells from human colorectal cancer.** (**a**) Perivascular cells from human colorectal cancer vessels were isolated and cultured. (**b**) Transmission electronic microscopy image of the primary cultured tumor perivascular cells, as characterized by large nuclei and few organelles. (**c,** **d**) Tumor perivascular cells were positive for perivascular cell markers, but negative for endothelial and epithelial markers. Perivascular cell surface markers were detected by (**c**) immunofluorescence assay and (**d**) flow cytometry analysis. DAPI (blue) staining indicated cell nuclei. Scale bar, 50 μm. Perivascular cell markers, α-SMA, FAPα, NG2, and PDGFRβ (red). Endothelial cell marker, CD31. Epithelial cell marker, E-cadherin.


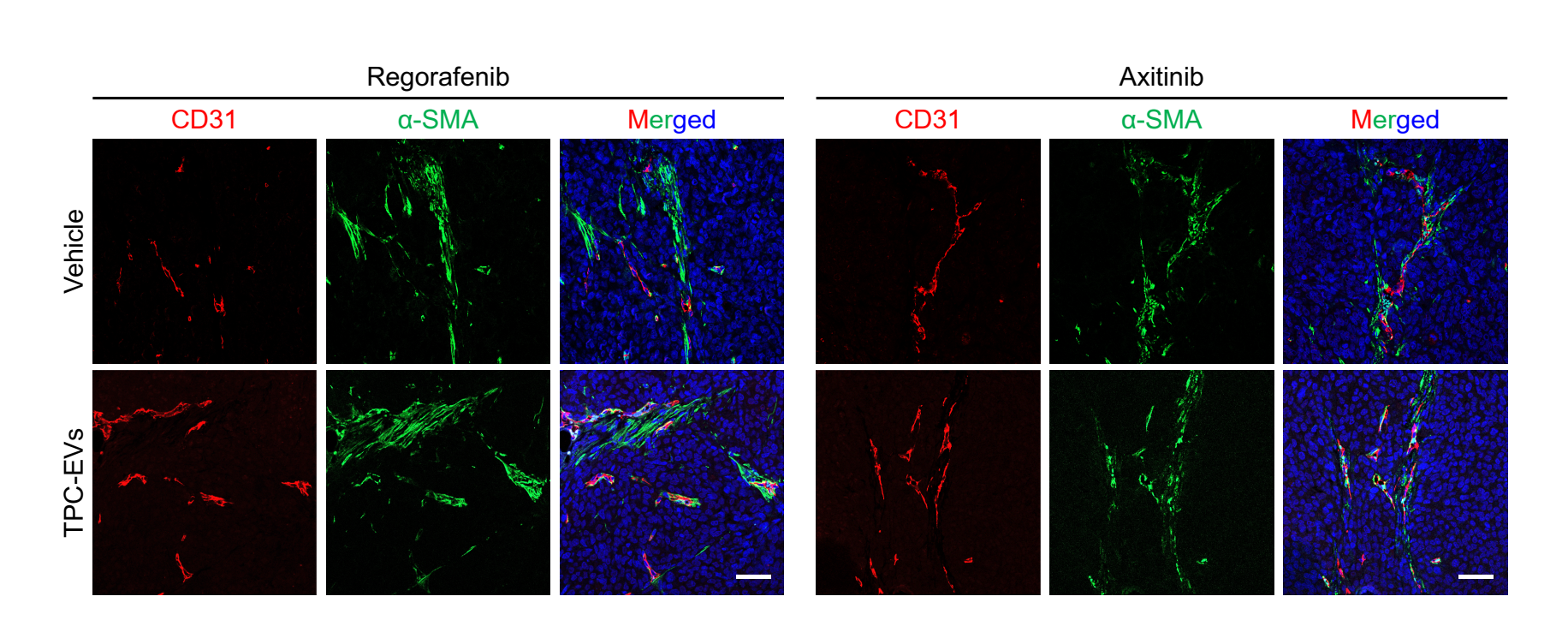


**Supplementary Figure 6.** **TPC-EVs promote revascularization in HCT116 tumors after treatment with AA-TKIs.** Representative images of CD31^+^ (red) endothelial cells, α-SMA^+^ (green) perivascular cell immunostaining, and DAPI (blue) for nuclear staining in tumors after treatment with Vehicle or TPC-EVs. Scale bar, 50 μm.


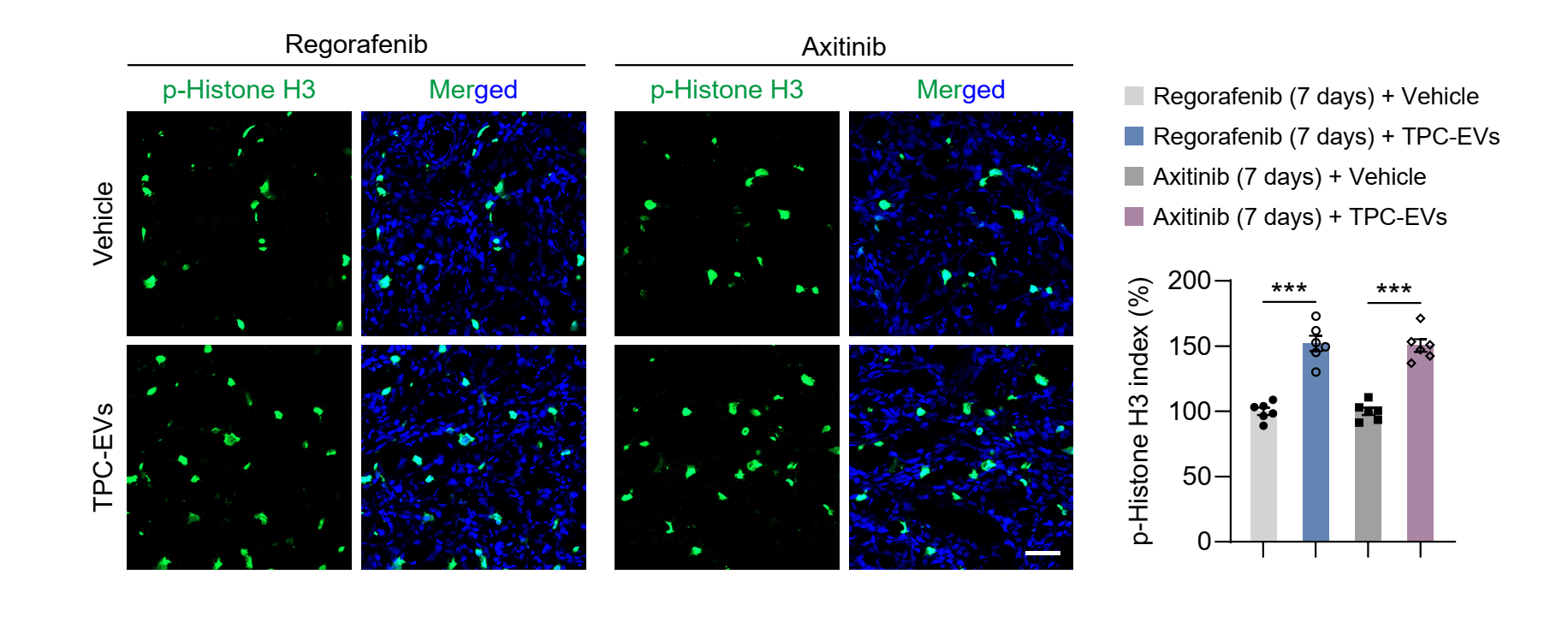


**Supplementary Figure 7.** **The proliferation of tumor cells is increased in TPC-EV-treated tumors.** Mice bearing HT-29 tumors were treated with regorafenib or axitnib for 7 day, followed by treatment with Vehicle or TPC-EVs for 4 days. Representative images of p-Histone H3 (green), and DAPI (blue) for nuclear staining in tumors after the indicated treatments. Scale bar, 50 μm. Quantification of cell proliferation (p-Histone H3 index) in tumors (n = 6). Data are present as mean ± SEM. ^***^*P* < 0.001.


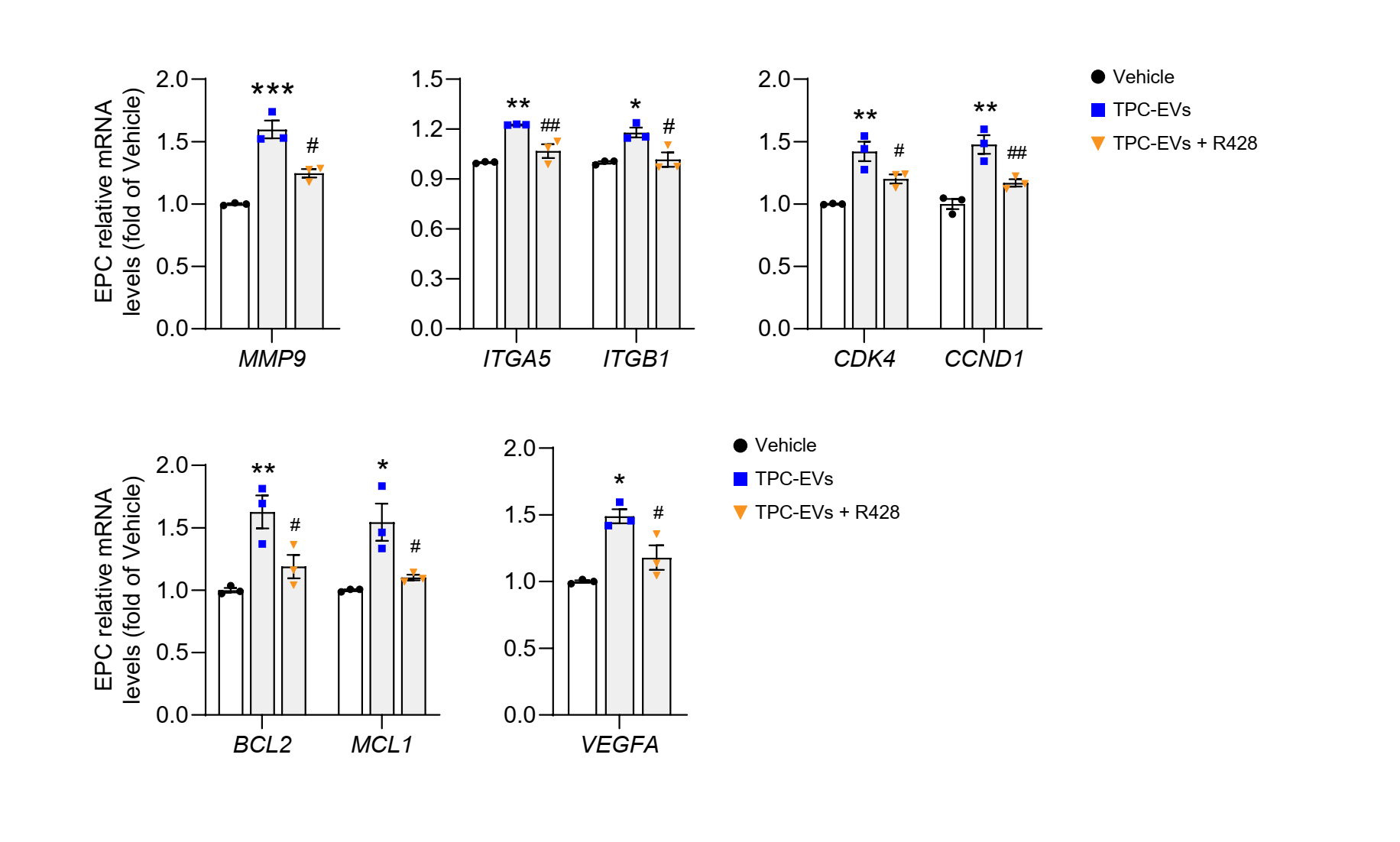


**Supplemental Figure 8. R428 treatment suppresses TPC-EV-induced increases in angiogenesis-related genes in EPCs.** EPCs were treated with TPC-EVs in the absence or presence of R428 for 24 h, and then total RNA was extracted and used for RT-PCR assay. Data are presented as mean ± SEM, n = 3. ^*^ *P* < 0.05, ^**^ *P* < 0.01, and ^***^ *P* < 0.001 versus the Vehicle groups. ^#^ *P* < 0.05, and ^##^ *P* < 0.01 versus the TPC-EV-treated groups. The *P* values were determined by one-way ANOVA and Tukey’s multiple comparison test.


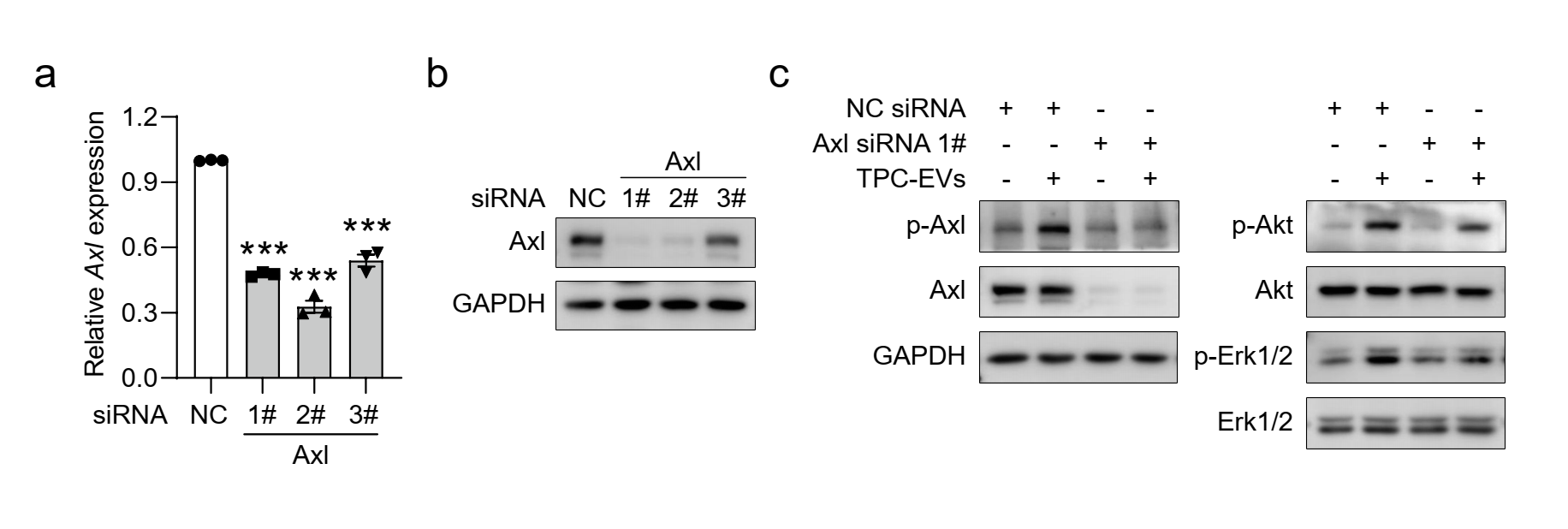


**Supplemental Figure 9. The knockdown of Axl inhibits TPC-EV-mediated activation of the Axl pathway in EPCs.** (**a**) Quantification of *Axl* mRNA level and (**b**) representative blots of EPCs after transfection with Axl siRNAs. (**c**) The phosphorylated and total forms of Axl, Akt, and Erk1/2 in transfected EPCs were determined by Western blotting assay. Data are presented as mean ± SEM, n = 3, ^***^*P* < 0.001 versus the EPCs transfected with NC siRNA.


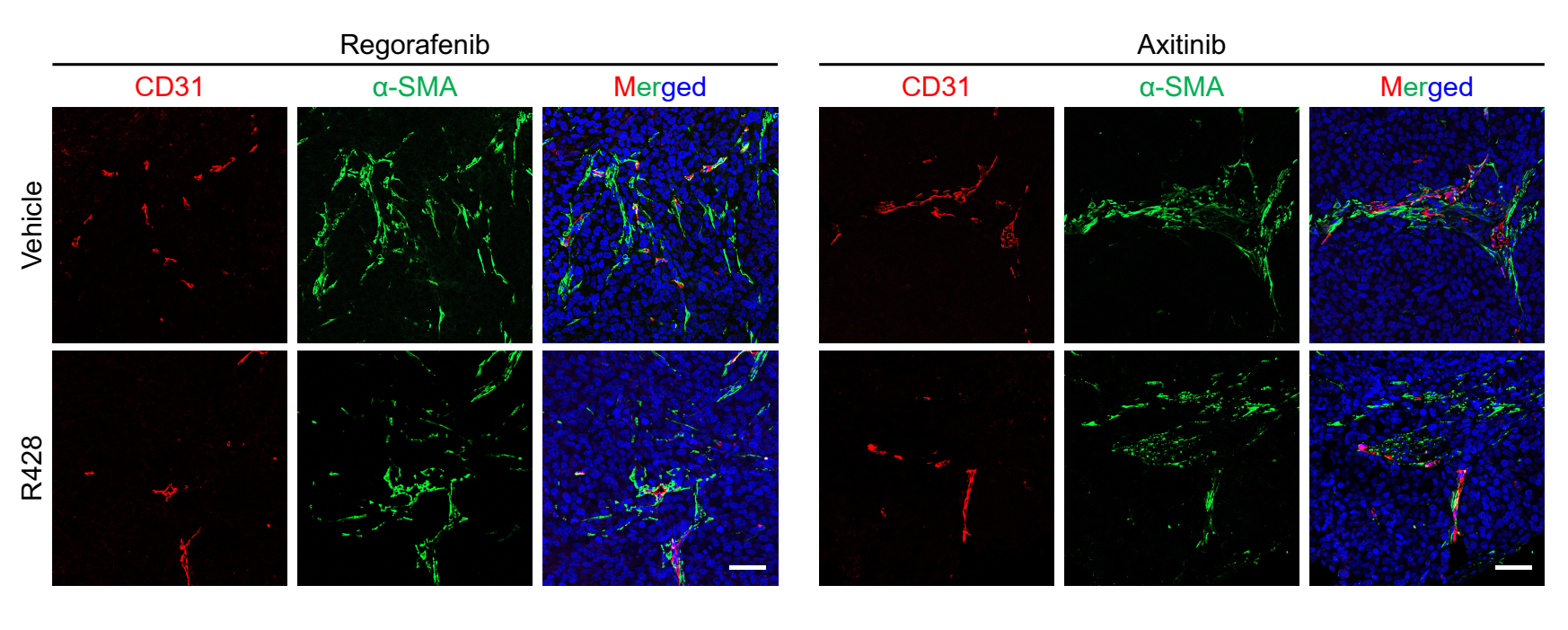


**Supplementary Figure 10.** **R428 treatment attenuates HCT116 tumor revascularization.** Representative images of CD31^+^ (red) endothelial cells, α-SMA^+^ (green) perivascular cell immunostaining, and DAPI (blue) for nuclear staining in tumors after the indicated treatments. Scale bar, 50 μm.

**Supplementary Table 1.** **Clinical characteristics of the resected cancers**

| Characteristics | Case 1 | Case 2 | Case 3 |
| --- | --- | --- | --- |
| Gender/Age (yr) | Female (61) | Male (58) | Male (51) |
| Date of diagnosis | 20190429 | 20190429 | 20190516 |
| Tumor type | Colorectal Adenocarcinoma | Colorectal Adenocarcinoma | Colorectal Adenocarcinoma |
| Location | Sigmoid colon | Rectum | Descending colon and ileum |
| Tumor size (maximum diameter) | 4 cm | 5 cm | 4 cm |
| Differentiation | Moderate | Moderate | Moderate |
| TNM stage | T4aN1M0 | T2N0M0 | T4bN1M1a |
| Clinical stage | IIIB | I | Ⅳa |
| Clinical metastasis | Lymph node metastasis | No metastasis | Liver metastasis |
| Treatment status | No treatment before surgery | No treatment before surgery | No treatment before surgery |

**Supplementary Table 2.** **Primer sequences used in RT-PCR assay**

| Gene name | Forward/Reverse | Sequence 5’ to 3’ |
| --- | --- | --- |
| *CDK4* | Forward | TACAAGGCCCGTGATCCCCA |
|  | Reverse | AGCCACCTCACGAACTGTGC |
| *CCND1* | Forward | CGTACCCCGATGCCAACCTC |
|  | Reverse | GCAGGCGGCTCTTTTTCACG |
| *BCL2* | Forward | GCACCGGGCATCTTCTCCTC |
|  | Reverse | CAGCTGGCTGGACATCTCGG |
| *MCL1* | Forward | GCCATCATGTCGCCCGAAGA |
|  | Reverse | GTCGTAAGGTCTCCAGCGCC |
| *MMP9* | Forward | CGCTCCTACTCTGCCTGCAC |
|  | Reverse | AGTCTCTCGCTGGGGCAGAA |
| *ITGA5* | Forward | GCCCCAGCTCCATTAGCCAG |
|  | Reverse | TCACAGCGCAGCCTGAAACA |
| *ITGB1* | Forward | ACCTGCCTTGGTGTCTGTGC |
|  | Reverse | CAGGATCAGGTTGGACCGGC |
| *VEGFA* | Forward | TCAGCGCAGCTACTGCCATC |
|  | Reverse | GTGCTGGCCTTGGTGAGGTT |
| *BCL2L2* | Forward | GTCGTAAGGTCTCCAGCGCC |
|  | Reverse | TCTCAGCACACAGTGCAGCC |
| *GAS6* | Forward | TGGCGCGGAATCTGGTCATC |
|  | Reverse | GGCTGCACGAGGTCCTTCTC |
| *AXL* | Forward | GAACCTTCAACTCCTGCCTTCTCG |
|  | Reverse | TTCATCGTCTTCACAGCCACCTTG |
| *ACTB* | Forward | TCTTCCAGCCTTCCTTCCTG |
|  | Reverse | CCTGCTTGCTGATCCACATC |

**Supplementary Table 3.** **SiRNA sequences used in cell transfection**

| Gene name | Sense/Antisense | Sequence 5’ to 3’ |
| --- | --- | --- |
| *GAS6*-homo-683 | Sense | GCCUCCAGAUCUGCCACAATT |
|  | Antisense | UUGUGGCAGAUCUGGAGGCTT |
| *GAS6*-homo-1443 | Sense | CCUGACCGUGGGAGGUAUUTT |
|  | Antisense | AAUACCUCCCACGGUCAGGTT |
| *GAS6*-homo-1857 | Sense | GCAUACGGCCUUGGCCCUATT |
|  | Antisense | UAGGGCCAAGGCCGUAUGCTT |
| *AXL*-homo-1950 | Sense | GCGGUCUGCAUGAAGGAAUTT |
|  | Antisense | AUUCCUUCAUGCAGACCGCTT |
| *AXL*-homo-1337 | Sense | GGACAUAGGGCUAAGGCAATT |
|  | Antisense | UUGCCUUAGCCCUAUGUCCTT |
| *AXL*-homo-1625 | Sense | GGAGACCCGUUAUGGAGAATT |
|  | Antisense | UUCUCCAUAACGGGUCUCCTT |
